# Epigenetic silencing of miR-296 and miR-512 ensures hTERT dependent apoptosis protection and telomere maintenance in basal-type breast cancer cells

**DOI:** 10.18632/oncotarget.21180

**Published:** 2017-09-23

**Authors:** Roberto Dinami, Valentina Buemi, Rosanna Sestito, Antonina Zappone, Yari Ciani, Miguel Mano, Eleonora Petti, Andrea Sacconi, Giovanni Blandino, Mauro Giacca, Silvano Piazza, Roberta Benetti, Stefan Schoeftner

**Affiliations:** ^1^ Laboratorio Nazionale Consorzio Interuniversitario Biotecnologie (LNCIB), Genomic Stability Unit, Trieste 34149, Italy; ^2^ Italian National Cancer Institute, Regina Elena, Rome 00144, Italy; ^3^ Department of Life Sciences, Università degli Studi di Trieste, Trieste 34127, Italy; ^4^ Laboratorio Nazionale Consorzio Interuniversitario Biotecnologie (LNCIB), Bioinformatics and Functional Genomics Unit (BFGU), Trieste 34149, Italy; ^5^ International Centre for Genetic Engineering and Biotechnology (ICGEB), Molecular Medicine Laboratory, Trieste 34149, Italy; ^6^ Italian National Cancer Institute, Regina Elena, Translational Oncogenomics Group, Rome 00144, Italy; ^7^ Laboratorio Nazionale Consorzio Interuniversitario Biotecnologie (LNCIB), Cancer Epigenetics Unit, Trieste 34149, Italy; ^8^ Department of Medical and Biological Sciences, Università degli Studi di Udine, Udine 33100, Italy

**Keywords:** miR-296-5p, miR-512-5p, telomerase, telomeres, breast cancer

## Abstract

The catalytic subunit of the telomerase complex, hTERT, ensures unlimited proliferative potential of cancer cells by maintaining telomere function and protecting from apoptosis. Using a miRNA screening approach we identified miR-296-5p and miR-512-5p as miRNAs that target hTERT in breast cancer cells. Ectopic miR-296-5p and miR-512-5p reduce telomerase activity, drive telomere shortening and cause proliferation defects by enhancing senescence and apoptosis in breast cancer cells. In line with the relevance of hTERT expression for human cancer we found that miR-296-5p and miR-512-5p expression is reduced in human breast cancer. Accordingly, high expression of miR-296-5p and miR-512-5p target genes including hTERT is linked with significantly reduced distant metastasis free survival and relapse free survival of basal type breast cancer patients. This suggests relevance of the identified miRNAs in basal type breast cancer. Epigenetic silencing of miR-296 and miR-512 encoding genes is responsible for low levels of miR-296-5p and miR-512-5p expression in basal type breast cancer cells. Disrupting gene silencing results in a dramatic upregulation of miR-296-5p and miR-512-5p levels leading to reduced hTERT expression and increased sensitivity to the induction of apoptosis. Altogether, our data suggest that epigenetic regulatory circuits in basal type breast cancer may contribute to high hTERT levels by silencing miR-296-5p and miR-512-5p expression, thereby contributing to the aggressiveness of basal type breast cancer.

## INTRODUCTION

Vertebrate telomeres consist of TTAGGG tandem repeats that are localized at chromosome ends and protect linear chromosomes from eliciting a DNA damage response [1, 2]. Telomeres are not completely replicated due to the “end replication problem” [1]. In embryonic tissues and stem cells, the reverse transcriptase telomerase replenishes telomere repeats at chromosome ends, thus resolving the end replication problem [3]. Absent or insufficient telomerase expression in primary and adult cells leads to progressive telomere shortening that finally causes to telomere dysfunction and the induction of senescence and apoptosis, thus representing a major tumor suppressive mechanism [4]. Consistent with this, reactivation of telomerase represents a hallmark feature in 80-90% of human cancers and confers replicative immortality [5]. The non-coding RNA component hTR and the catalytic subunit hTERT are essential for telomere maintenance by telomerase [6]. Remarkably, the tumor promoting effect of hTERT is not limited to telomere maintenance. In fact, hTERT has been found to promote DNA repair, interferes with key pathways of tumor formation and protects cells from mitochondrial apoptosis [7–16]. Accordingly, hTERT transcription is controlled by central pathways in tumorigenesis and tumor suppression [17–20]. In addition to classic transcriptional regulation, the acquisition of activating histone modification and defined DNA methylation pattern result in the activation of the hTERT promoter in cancer cells [21–23]. Micro RNAs (miRNAs) were demonstrated to control the expression of crucial tumor suppressors or oncogenes and critically impact on hallmark features of human cancer [24]. Accordingly, cancer type specific miRNA expression signatures have been established as efficient prognostic and predictive biomarkers in diverse types of human cancer [24]. Recent studies showed that miRNAs can control telomere function by targeting the expression of telomere regulators [25, 26]. Several miRNAs were shown to target hTERT resulting in reduced proliferation of cells derived from anaplastic thyroid carcinoma, neuroblastoma, gastric cancer, cervical cancer, head and neck squamous cell carcinoma and leukemic T-cell lymphoblasts [27–33]. Our study focuses on the identification of miRNAs that target hTERT and have relevance for human breast cancer. Performing a high-throughput luciferase reporter screen we show that miR-296-5p and miR-512-5p efficiently target the 3’UTR of hTERT. Both miRNAs are donwn-regulated in breast cancer. Gene expression analyses suggest that high expression of hTERT and a panel of cancer relevant miR-296-5p and miR-512-5p target genes appear to be linked with reduced distant metastasis free survival and relapse free survival of basal breast cancer patients. Ectopic miR-296-5p and miR-512-5p reduce telomerase activity, impair telomere maintenance and promote senescence and apoptosis in basal breast cancer cells. We further show that miR-296 and miR-512 gene loci are subjected to gene silencing in basal type breast cancer cells. Disrupting miRNA gene silencing by the use of epigenetic drugs causes a dramatic miR-296-5p and miR-512-5p upregulation and concomitant reduction of hTERT expression that reduces the resistance to apoptotic stimuli. This suggests that miR-296-5p and miR-512-5p execute epigenetic programs that control hTERT expression in breast cancer cells.

Altogether, our data suggest that silencing of miR-296-5p and miR-512-5p in basal type breast cancer helps to establish high hTERT expression levels, and may contribute to basal type breast cancer aggressiveness and reduced patient survival.

## RESULTS

### miR-296-5p and miR-512-5p target the 3’UTR of hTERT and are down-regulated in breast cancer

**Figure 1:**
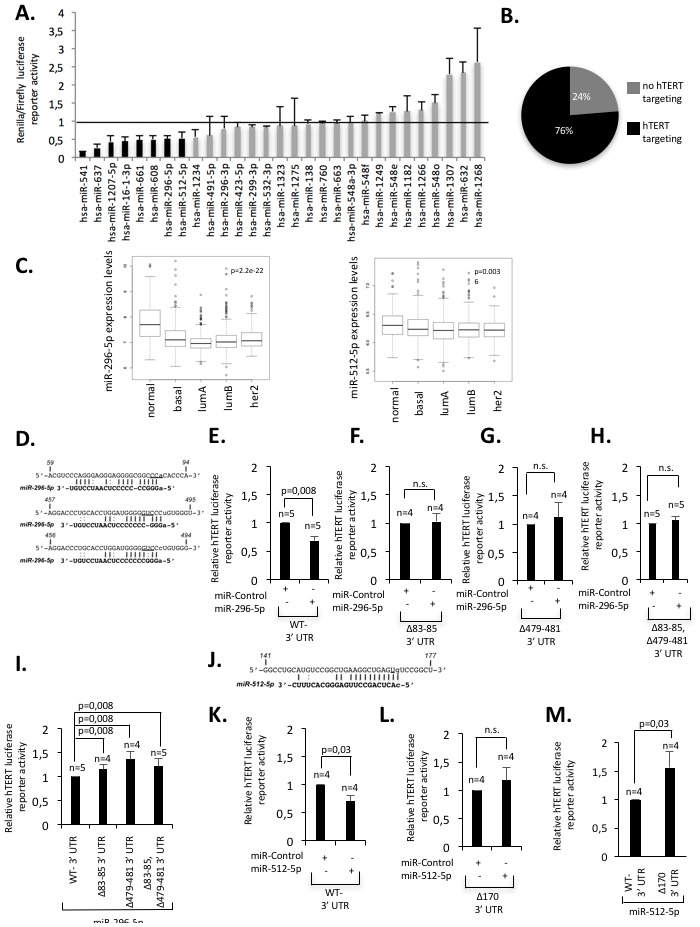
Identification of miRNAs that target hTERT in human breast cancer **(A)** Results of the luciferase reporter screen. Renilla:Firefly luciferase ratios of individual candidate miRNAs are shown. Luciferase reporter ratio of a control miRNA was set “1”. Luciferase reporter ratios <1 indicate target specify of candidate miRNAs for the 3’UTR of hTERT. Candidate miRNAs with ratios <0.5 were considered for further analysis. Experiments were carried out in duplicate; standard deviation is shown by error bars. **(B)** Proportion of candidate miRNAs that reduced hTERT-3’UTR luciferase reporter activity **(C)** miR-296-5p and miR-512-5p expression in normal breast tissue and human breast cancer subtypes using a miRNA expression dataset [34]. Expression values are shown in box blots at a log2 scale; a Wilcox test was used to calculate the indicated p-values (see also Supplementary figure legend 2A and Material and methods). **(D)** Schematic representation of miR-296-5p target sites in the 3’UTR of hTERT; underlined nucleotides indicate the position of deletions in mutant hTERT 3’UTR constructs. **(E-H)** Luciferase reporter assays in MDA-MB-231 cells using wild type hTERT 3’UTR constructs (E) or constructs that contain mutations of the respective miR-296-5p target sites (F-H). Mutations of miR-296-5p target sites result in increased luciferase reporter activity, when compared to the hTERT wild-type 3’UTR reporter constructs. **(I)** Luciferase reporter assays using MDA-MB-231 cells co-transfected with mimic-miR-296-5p and the indicated hTERT 3’UTR luciferase reporters. Mutations in the hTERT 3’UTR increase luciferase reporter activity. **(J)** Schematic representation of miR-512-5p target sites in the 3’UTR of hTERT; underlined nucleotides indicate the position of deletions in mutant hTERT 3’UTR constructs **(K, L)** Luciferase reporter assays in MDA-MB-231 cells using a wild type hTERT 3’UTR constructs (K) or a construct that contains a mutations of the predicted miR-512-5p target sites (L). **(M)** Luciferase reporter assays using MDA-MB-231 cells co-transfected with mimic-miR-512-5p and the indicated hTERT 3’UTR luciferase reporters. Mutations in the hTERT 3’UTR increase luciferase reporter activity. n, number of independent experiments; error bars show standard deviation, E-M.: p values were calculated using a Mann Whitney test; n.s., non significant - p-value >0,05.

Identifying pathways that limit telomere function or telomerase activity in human cancer can reveal potential targets for cancer therapies. We used miRNA target prediction software to establish a list of miRNA candidates with predicted target specificity for the 3’UTR of hTERT and subsequently performed a candidate mimic-miRNA siRNA luciferase reporter screen for candidate validation [26] (Materials and Methods). Hela cells were transiently co-transfected with candidate mimic-miRNA siRNAs and a luciferase reporter vector, that contained i) the Renilla luciferase cDNA fused to the full length hTERT 3’UTR (561 nucleotides) and ii) an independent Firefly luciferase expression cassette to correct for transfection efficiency (Supplementary Figure 1A). Three days post-transfection, Renilla to Firefly luciferase luminescence ratios were determined by dual-luminometry (Figure 1A, Supplementary Figure 1B). We found that 76% of candidate miRNAs showed reduced Renilla:Firefly luminescence values (<1) when compared to non-specific control mimic-miRNA siRNAs (Control mimics set “1”; Figure 1B). This result confirms the specificity of *in-silico* target prediction analysis. Recent studies reported a role of miR-133a, miR-138, miR-541, miR-491-5p, miR-512-5p, miR-1182, miR-1207-5p and miR-1266 in the control of hTERT expression in various types of cancer cells [27–33]. In our screen we identified miR-296-5p as novel hTERT-targeting miRNA. Amongst miRNAs with reported targeting specificity for hTERT, miR-541, miR-512-5p and miR-1207-5p were able to efficiently reduce hTERT-3’UTR reporter activity in our screen; miR-133a was not represented in our candidate miRNAs list (Supplementary Figure 1B). Absence of targeting specificity for a subset of miRNAs with reported hTERT targeting specificity may be attributable to cell type specific effects that may impact on targeting efficiency. With this study we aimed to extend knowledge on miRNA dependent regulation of telomere function in the context of breast cancer. We thus focused functional analysis of candidate miRNAs on this cancer type [26]. We selected miRNAs for further analysis that i) mediate at least 50% reduction of luciferase reporter activity and ii) show altered expression in a miRNA expression dataset containing 1,302 breast tumors with detailed clinical annotation [34]. Given that hTERT re-expression is critical for cellular immortalization we hypothesized that functionally relevant miRNAs are down-regulated in breast cancer, thus facilitating improved telomere maintenance and protection from apoptosis. We found that among candidate miRNAs that mediate at least 50% reduction of hTERT 3’UTR reporter activity, only miR-296-5p, miR-512-5p and miR-1207-5p showed significant down-regulation in breast cancer when compared to healthy tissue (Figure 1A, 1C, Supplementary Figure 2A). Expression levels of miR-16-1^*^, miR-541, miR-637, miR-661 or miR-608 are not altered in breast cancer tissue (Supplementary Figure 2A). miR-512-5p and miR-296-5p have a reported role in various aspects of human cancer. In particular, miR-296-5p was demonstrated to have a tumor suppressive role in breast, prostate, non-small cell lung cancer or glioblastoma [35–40]. miR-512-5p was reported to activate apoptotic pathways in lung and gastric cancer and target hTERT in head and neck squamous cell carcinoma [32, 41–43]. We therefore focused our further study on the functional relevance of miR-296-5p and miR-512-5p in controlling hTERT expression in human breast cancer.

### miR-512-5p and miR-296-5p regulate telomere homeostasis by targeting hTERT expression

The 3’UTR of hTERT contains 3 predicted target sites for miR-296-5p and one target site for miR-512-5p (Figure 1D, 1J). miR-296-5p–hTERT target sites are conserved in humans, chimpanzee and rhesus monkeys (Supplementary Figure 2B). In contrast, miR-512-5p - hTERT target site interaction is limited to humans and chimpanzee (Supplementary Figure 2B). Remarkably, the extremely short hTERT 3’UTR in rodents does not contain miR-296-5p and miR-512-5p target sites (data not shown). This suggests different evolution of miRNA dependent regulatory pathways that control hTERT expression in vertebrate species. To validate the specificity of miR-296-5p and miR-512-5p for the 3’UTR of hTERT we generated hTERT 3’UTR luciferase reporter constructs that contain deletions of the individual miR-296-5p or miR-512-5p target sites (Figure 1D, 1J). We found that Hela cells transiently transfected with hTERT 3’UTR luciferase reporters carrying mutations in individual target sites for miR-296-5p or miR-512-5p show increased luciferase reporter activity when compared to cells transfected with a wild type 3’UTR luciferase reporter (Supplementary Figure 2C, 2D). These data support evidence for a link between miR-296-5p and miR-512-5p and the regulation of hTERT expression. A series of luciferase reporter experiments were carried out in HeLa and MDA-MB-231 basal type breast cancer cells that display comparable expression levels of endogenous miR-296-5p or miR-512-5p, respectively (Supplementary Figure 3A-3B). We found that ectopic introduction of mimic-miR-296-5p efficiently reduced luciferase reporter activity in both, MDA-MB-231 and HeLa cells when compared to control mimic-miRNA transfected cells (Figure 1E, Supplementary Figure 3C). Deleting individual miR-296-5p target sites present in the 3’UTR of hTERT renders the hTERT 3’UTR luciferase reporter resistant to ectopically increased miR-296-5p levels (Figure 1F-1H, Supplementary Figure 3D-3F). Importantly, MDA-MB-231 cells transiently co-transfected with mimic-miR-296-5p and mutant hTERT 3’UTR constructs displayed significantly increased luciferase activity when compared with the wild-type reporter (Figure 1I). Introducing deletions that disrupt the miR-512-5p target site in the 3’UTR of hTERT caused increased luciferase reporter activity in mimic-miR-512-5p transfected Hela and MDA-MB-231 cells when compared to the wild-type reporter constructs (Figure 1J-1L, Supplementary Figure 3G-3H). Finally, MDA-MB-231 cells transiently co-transfected with mimic-miR-512-5p and mutant hTERT 3’UTR constructs displayed significantly increased reporter activity when compared with cells co-transfected with mimic-miR-512-5p and the wild-type reporter (Figure 1M). In line with gain of function experiments, competing endogenous miR-296-5p or miR-512-5p by transfecting MDA-MB-231 cells with antagomiR siRNAs increased hTERT 3’UTR luciferase activity (Supplementary Figure 3I). Together, these data demonstrate target specificity of miR-296-5p and miR-512-5p for the 3’UTR of hTERT.

**Figure 2:**
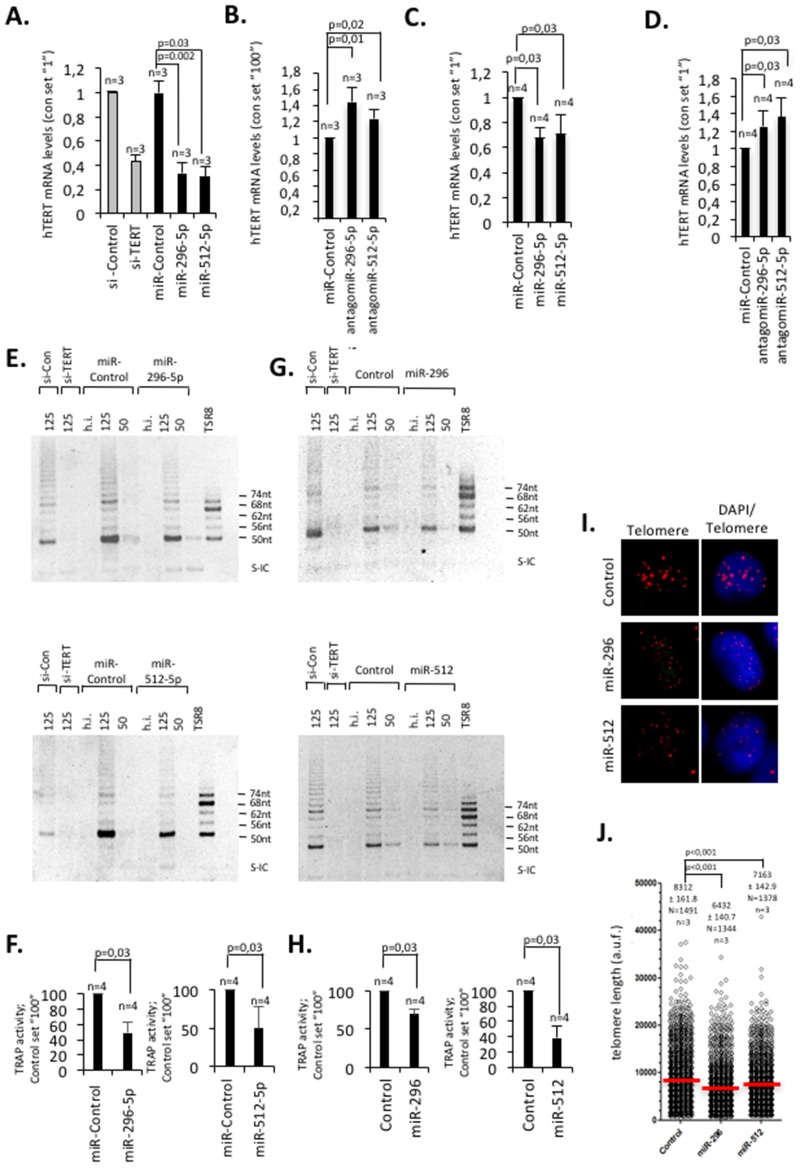
miR-296-5p and miR-512-5p act as negative regulators of telomerase activity and telomere length by targeting hTERT expression **(A, C)** Transient transfection of MCF-7 (A) or MDA-MB-231 (C) cells with hTERT specific siRNAs or miR-296-5p or miR-512-5p mimics causes a reduction of hTERT mRNA expression as determined by quantitative RT-PCR. Actin was used as reference mRNA. **(B, D)** Transient transfection of MCF-7 (B) or MDA-MB-231 (D) cells with antagomiR-296-5p or antagomiR-512-5p causes an increase of hTERT mRNA expression as determined by quantitative RT-PCR. Actin was used as reference mRNA. **(E)** Telomerase activity in lysates of MCF-7 transiently transfected with the indicated siRNAs or mimic-miRNA-siRNAs, as determined by the TRAP assay. Numbers (125, 50) indicate the amount of protein used for the assay (ng). **(G)** Telomerase activity in lysates of MDA-MB-231 transduced with a retroviral vector containing a miR-296 or miR-512 mini-gene cassette, as determined by the TRAP assay. Numbers (125, 50) indicate the amount of protein used for the assay (ng) **(F, H)** Quantification of TRAP assay in MCF-7 (F) or MDA-MB-231 (H) cells: ectopic miR-295-5p and miR-512-5p significantly reduce telomerase activity. **(I, J)** Quantitative telomere DNA FISH of MDA-MD-231 cells overexpressing miR-296 or miR-512 minigenes, respectively [70]. Control refers to an empty vector control. N, number of telomeres analyzed in DNA-FISH experiments. n, number of independent experiments; error bars show standard deviation, a.u.f., arbitrary fluorescence units; red line shows average telomere length; h.i., heat inactivated; S-IC, internal control PCR product; TSR8 control template for PCR. A-G, p values were calculated using a Mann Whitney test; n.s., non significant - p-value >0,05. I-J, an unpaired students t-test was used to calculate statistical significance.

We next wished to demonstrate that miR-296-5p and miR-512-5p impact on hTERT expression in classic breast cancer model cell lines. Ectopic introduction of mimic-miR-296-5p or mimic-miR-512-5p in MCF-7 luminal type or MDA-MB-231 basal type breast cancer cells caused an approximately 70% or 30% reduction of endogenous hTERT mRNA expression levels, respectively (Figure 2A, 2C). Of notice, ectopic introduction of miR-296-5p in MDA-MB-231 cells did not alter miR-512-5p levels; altered miR-512-5p levels did not change miR-296-5p levels. This does not support a cross-talk between these miRNAs (data not shown). Introduction of antagomiR-296-5p or antagomiR-512-5p that target endogenous mature miR-296-5p or miR-512-5p, resulted in a significant increase of hTERT mRNA expression levels in both, MCF-7 and MDA-MB-231 cells (Figure 2B, 2D). Together, this gives strong evidence that in breast cancer cells hTERT expression levels is directly controlled by miR-296-5p and miR-512-5p dosage. However, given the fact that multiple pathways impinge on telomerase expression, indirect effects triggered miR-296-5p or miR-512-5p on altered hTERT expression cannot be completely excluded. To test whether miR-296-5p or miR-512-5p dependent reduction of hTERT mRNA levels is paralleled by a reduction of telomerase activity we performed TRAP analysis. In accordance with hTERT mRNA expression data we found that ectopic introduction of miR-296-5p or miR-512-5p resulted in a significant reduction of overall telomerase activity in MCF-7 cells; this result was recapitulated by siRNA mediated knock down of hTERT (Figure 2E-2F). In line with this, stable expression of a miR-296-5p or miR-512-5p precursor stemloop construct in MDA-MB-231 basal type breast cancer cells, resulted in a significant reduction of hTERT expression and telomerase activity after approximately 18 population doublings. (Figure 2G, 2H; Supplementary Figure 4A-4C). This effect was paralleled by a shortening of telomere length, as determined by quantitative telomere DNA-FISH analysis (Figure 2I, 2J). Consistent with an overall decrease in telomere length we found that the frequency of short telomeres increases whereas the frequency of long telomeres decreases in miR-296-5p or miR-512-5p overexpressing MDA-MB-231 cells (Supplementary Figure 4D). The same telomere phenotype was found in MDA-MD-231 cells stably overexpressing a short hairpin RNA construct targeting hTERT (Supplementary Figure 4E-4H). Together, this indicates that miR-296-5p and miR-512-5p are regulators of telomerase expression that impact on telomerase activity and telomere homeostasis in breast cancer cells.

### Increased hTERT and miR-296-5p/miR-512-5p target gene expression is linked to poor clinical outcome in basal type breast cancer

**Figure 3:**
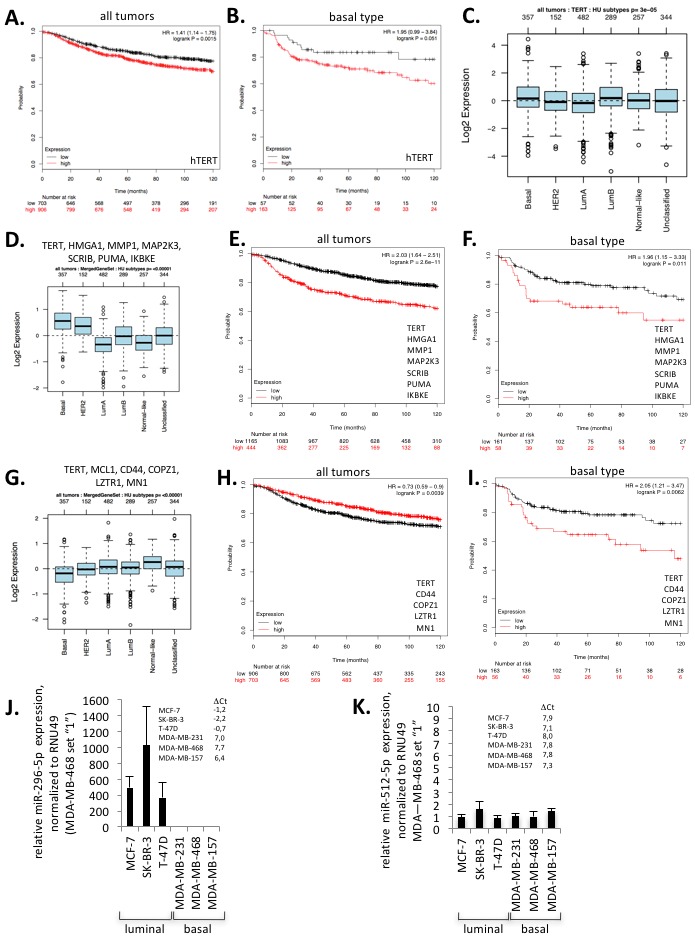
miR-296-5p and miR-512-5p contribute to poor survival **(A, B)** Kaplan–Meier survival curve considering distant metastasis free survival and relapse free survival (DMSF_mixed) using KM plotter across all types of breast cancer types (A) or basal type breast cancer (B) [44]. Cancer specimens were classified into hTERT high or low expression (Material and methods section). **(C)** Box-plots showing hTERT expression in various breast cancer types; p indicates ANOVA test significance. **(D)** Box-plots showing the expression of miR-296-5p target genes, previously validated in MDA-MB-231 cells. **(E, F)** Kaplan–Meier survival curve of time considering both distant metastasis free survival and relapse free survival (DMSF_mixed) across all types of breast cancer types (E) or basal type breast cancer (F). Cancer specimens with high expression of miR-296-5p target genes, previously validated in MDA-MB-231 cells, show poor survival. **(G)** Box-plots showing the expression of miR-512-5p target genes previously validated in MDA-MB-231 cells. **(H, I)** Kaplan–Meier survival curve considering both distant metastasis free survival and relapse free survival (DMSF_mixed) across all types of breast cancer types (H) or basal type breast cancer (I). Basal type breast cancer patients showing high expression of experimentally validated miR-512-5p target genes show poor survival. **(J)** miR-296-5p expression in basal type and luminal type breast cancer cell lines, as determined by quantitative TaqMan RT-PCR. miR-296-5p levels were quantified against RNU49. ΔCt values (Ct miR-296-5p – Ct RNU49) are indicated. **(K)** miR-512-5p expression in basal type and luminal type breast cancer cell lines, as determined by quantitative TaqMan RT-PCR. miR-512-5p levels were quantified against RNU49. ΔCt values (Ct miR-512-5p – Ct RNU49) are indicated. J, K.: 3 independent experiments were carried out, error bars indicate statistical significance; to better visualize differences in miR-296-5p and miR-512-5p, respective expression levels were set “1” in MDA-MB-468 cells.

We next set out to investigate whether hTERT expression and miR-296-5p or miR-512-5p target gene expression signatures impact on clinical parameters of defined subtypes of breast cancer. Kaplan-Meier survival curves analyzing two independent datasets, comprising 4142 or 1881 breast cancer samples revealed that increased hTERT expression leads to significantly reduced distant metastasis free survival and relapse free survival when pooling all human breast cancer subtypes (Figure 3A, Supplementary Figure 5A) [44, 45]. Remarkably, this effect is exacerbated in basal type breast cancer where hTERT expression performs as independent predictor of poor prognosis (Figure 3B; Supplementary Figure 5B, 5C). Importantly, hTERT expression does not impact on distant metastasis free survival and relapse free survival in other breast cancer subtypes such as luminal A, luminal B, estrogen receptor positive, estrogen receptor negative, Erb2 positive or normal-like breast cancer (Supplementary Figure 5D-5I). This result is also supported by an elevated hTERT expression in basal type breast cancer (Figure 3C). Together, this suggests that mechanisms that regulate hTERT expression have increased relevance in basal type breast cancer. We next used public gene expression datasets to test whether the expression of validated miR-296-5p or miR-512-5p target genes may have a special relevance in basal type breast cancer. Recent studies demonstrate targeting of HMGA1, MMP1, MAP2K3 and SCRIB by miR-296-5p in MDA-MB-231 cells [35, 36]. In addition, we confirmed down-regulation of previously reported miR-296-5p targets IKBKE and PUMA (BBC3) in mimic-miR-296-5p transfected MDA-MB-231 cells (Supplementary Figure 6A-6E). We found that the expression of miR-296-5p target genes hTERT, HMGA1, MMP1, MAP2K3, SCRIB, PUMA (BBC3) and IKBKE is higher in basal breast cancer and linked with significantly reduced distant metastasis free survival and relapse free survival in pooled breast cancer subtypes and basal type breast cancer (Figure 3D, 3F). Altered expression of miR-296-5p target genes does not impact on patient survival of other breast cancer subtypes (data not shown). Additional validation experiments revealed that out of a panel of predicted and reported miR-512-5p target genes only COPZ1, MN1 and LZTR1 are down regulated after ectopic introduction of mimic-miR-512-5p in MDA-MB-231 cells (Supplementary Figure 7A-7H). The expression of these genes does not show strong variation between different breast cancer types and slightly improves distant metastasis free survival and relapse free survival in pooled breast cancer subtypes (Figure 3G, 3H). However, distant metastasis free survival and relapse free survival appears to be significantly reduced in basal type breast cancer with increased miR-512-5p target gene expression (Figure 3I). A similar trend for miR-296-5p and miR-512-5p target genes was found in the GOBO dataset (Supplementary Figure 6F-6H; Supplementary Figure 7I-7K)[45]. Together, these data may provide indications that miR-296-5p and miR-512-5p dependent regulation of hTERT is part of a miR-296-5p/miR-512-5p target gene signature that contributes to poor clinical outcome in basal type breast cancer. This data is supported by down-regulation of miR-296-5p and miR-512-5p in basal type human breast cancer (Figure 1C). We consequently focused further experiments on basal type breast cancer. Expression analysis revealed that miR-296-5p expression levels are dramatically reduced in a panel of basal type breast cancer cell lines including MDA-MB-231, MDA-MB-468 and MDA-MB-157 when compared to luminal type breast cell lines MCF-7, SK-BR3 or T-47D (Figure 3J). Remarkably, human mammary epithelial cells display increased miR-296-5p expression levels compared to basal type breast cancer cells (Supplementary Figure 7L). This underlines that down-regulation of miR-296-5p represents a general feature of basal-type breast cancer cells. miR-512-5p/RNU46 ΔCt values of Taq-man PCR miRNA expression analysis indicate that miR-512-5p levels are close to the detection limit in luminal and basal type breast cancer cells but also in human mammary epithelial cells (Figure 3K, Supplementary Figure 7M). Thus, low miR-512-5p expression is a general feature of primary breast epithelial but also breast cancer cells.

Altogether our data suggest that low miR-512-5p and miR-296-5p expression may have a role in assisting the establishment of a gene expression signature that includes hTERT upregulation, and contributes to the aggressiveness of basal type breast cancer.

### miR-296-5p and miR-512-5p cause hTERT dependent cell proliferation defects in MDA-MB-231 basal type breast cancer cells

**Figure 4:**
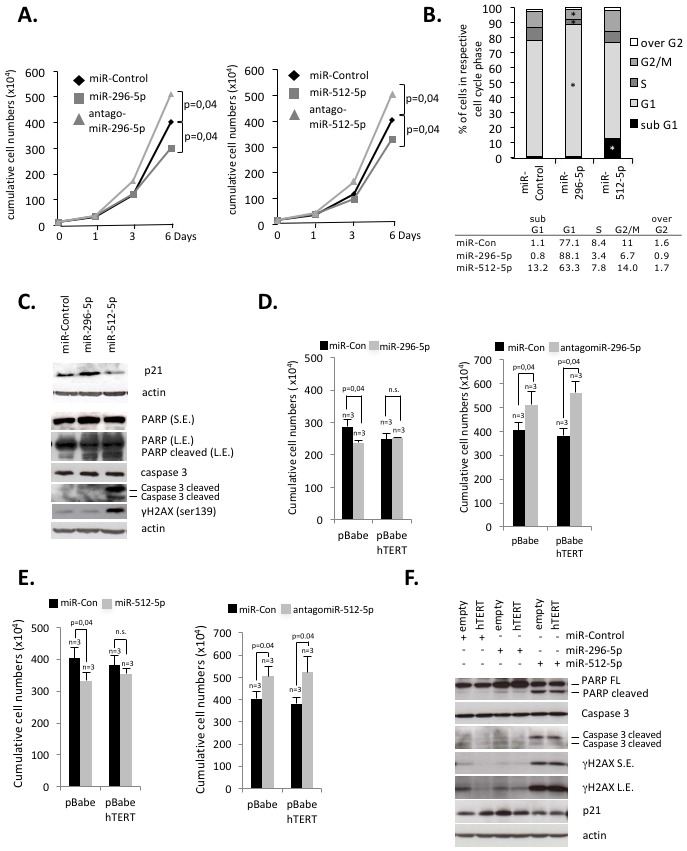
miR-296-5p and miR-512-5p suppress hTERT-mediated protection from apoptosis and senescence in basal type breast cancer cells **(A)** Cumulative cell numbers of MDA-MB-231 cells transiently transfected with miRNA control, miR-296-5p or miR-512-5p mimics. miR-296-5p and miR-512-5p reduce cell proliferation. Respective antagomiR siRNAs improve MDA-MB-231 cell proliferation. Cells were transfected at day 0 and day 3 of the experiment. **(B)** FACS cell cycle profile of MDA-MB-231 cells treated with the indicated mimic-miRNA-siRNAs. Cells were treated as described in (A). Top panel, percentage of cells in the respective cell cycle phases; bottom panel, numeric results of shown in the top panel. Experiments were carried out in triplicate, average values are shown. **(C)** Western blotting of apoptosis, DNA damage and proliferation markers. Actin was used as a loading control. **(D)** Ectopic hTERT expression from a retroviral vector rescues proliferation defects of miR-296-5p transfected MDA-MB-231 cells. Transfection with antagomiR-296-5p improves proliferation. Cells were transfected at day 0 and day 3 of the experiment; cumulative cell numbers were determined at day 6. **(E)** Ectopic hTERT expression from a retroviral vector rescues proliferation defects of miR-512-5p transfected MDA-MB-231 cells. Transfection with antagomiR-512-5p improves proliferation. Cells were transfected at day 0 and day 3 of the experiment; cumulative cell numbers were determined at day 6. **(F)** Western blotting of MDA-MB-231 cells, treated as described in (D) and (E). Ectopic hTERT expression rescues proliferation, DNA damage and apoptosis marker expression triggered by miR-296-5p or miR-512-5p. Actin was used as a loading control. n, number of independent experiments; error bars show standard deviation, p values were calculated using a Mann Whitney test; n.s., non significant - p-value >0,05. ^*^ indicates a p-value <0,05. FL, full length; S.E., short exposure; L.E., long exposure.

In order to test the impact of miR-296-5p and miR-512-5p on cell proliferation we determined cumulative cell numbers of MDA-MB-231 basal type breast cancer cells with altered miR-296-5p and miR-512-5p levels. We found that ectopic introduction of synthetic miR-296-5p or miR-512-5p causes a significant reduction of MDA-MB-231 cell proliferation (Figure 4A). In line with this, targeting of endogenous miR-296-5p or miR-512-5p using antagomiRs increased cell proliferation rates (Figure 4A). Cell cycle FACS analysis revealed that introduction of synthetic miR-296-5p siRNAs caused increased cell numbers in G1 phase and reduced cell numbers in S and G2/M phase, indicative for a G1-S phase arrest (Figure 4B). In contrast, ectopic miR-512-5p causes a significant increase in subG1, indicative for the activation of apoptosis (Figure 4B). This result was confirmed by western blotting showing that ectopic introduction of synthetic miR-512-5p results in increased levels of the DNA damage marker γH2AX and increased PARP and Caspase 3 cleavage (Figure 4C). Ectopic introduction of miR-296-5p in MDA-MB-231 cells mediates increased p21 protein levels, modest PARP cleavage, as well as increased numbers of beta-galactosidase positive cells (Figure 4C; Supplementary Figure 8A). This indicates that miR-296-5p promotes cellular senescence but also supports a modest activation of apoptosis programs. In contrast, ectopic miR-512-5p did not increase number of beta-galactosidase positive cells, thus selectively promoting apoptosis (Supplementary Figure 8A). A prominent role of miR-512-5p in activating apoptosis programs in MDA-MD-231 cells was validated by FACS analysis of Annexin V staining (Supplementary Figure 8B). Analogous data were obtained using p53 proficient MCF-7 luminal breast cancer cells: introducing miR-296-5p mimics into luminal MCF-7 cells activated senescence and apoptosis pathways; ectopic miR-512-5p exclusively activated apoptosis (Supplementary Figure 8C, 8D). In line with this, miR-296-5p and miR-512-5p have an anti-proliferative function in MCF-7 cells (Supplementary Figure 8E). We next validated that impaired proliferation of miR-296-5p or miR-512-5p transfected MDA-MB-231 basal type breast cancer cells is due to reduced hTERT expression. For this reason we transduced MDA-MB-231 cells with retroviruses encoding the human hTERT cDNA (Supplementary Figure 8F). Importantly, we found that ectopic expression of hTERT rescues miR-296-5p or miR-512-5p induced cell proliferation defects (Figure 4D and 4E). In line with this, ectopic hTERT reduced p21 expression in miR-296-5p transfected cells and reduced γH2AX levels as well as PARP and Caspase 3 cleavage in miR-296-5p or miR-512-5p transfected cells (Figure 4F). As expected, transfection of MDA-MB-231 cells with antagomiR-296-5p or antagomiR-512-5p improved MDA-MB-231 cell proliferation (Figure 4D, 4E, right panels). This suggests that direct targeting of the hTERT 3’UTR by miR-512-5p or miR-296-5p has a major contribution to the observed proliferation defects. Together, these data show that low expression of miR-296-5p and miR-512-5p ensures elevated hTERT expression to improve cell proliferation potential.

### Epigenetic silencing of miR-296-5p and miR-512-5p protects MDA-MB-231 cells from apoptosis

**Figure 5:**
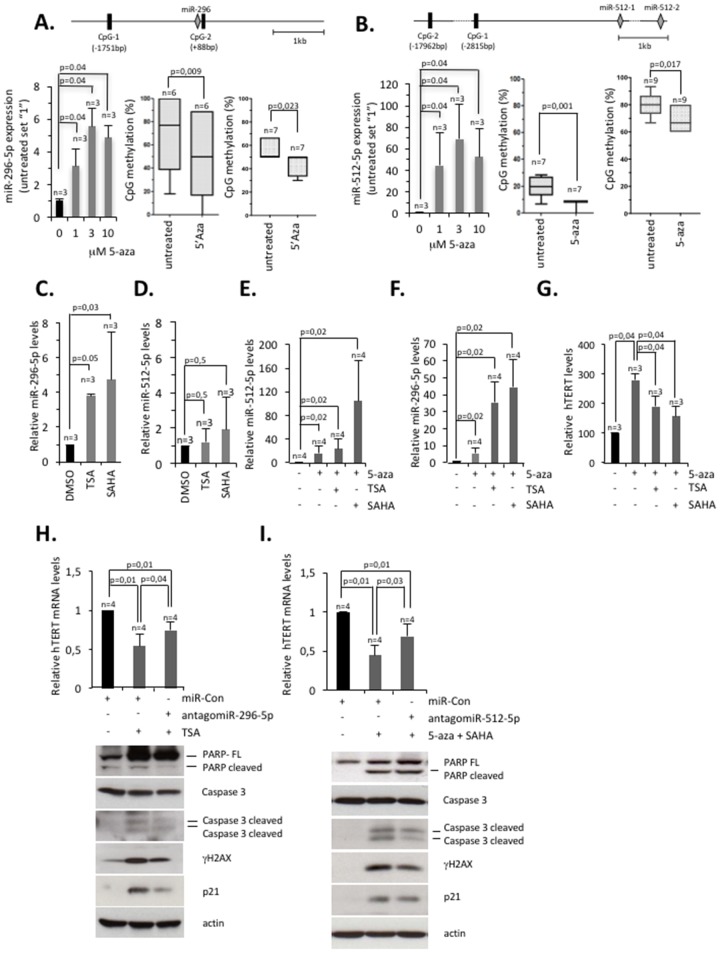
Epigenetic silencing of miR-296-5p and miR-512-5p ensures hTERT expression in basal-type breast cancer cells **(A)** miR-296-5p expression upon inhibition of DNA methylation as determined by quantitative TaqMan RT-PCR. Expression levels were normalized against RNU49. Top panel, Positions of CpG islands analyzed for the miR-296 locus. Numbers indicate distance from miR-296. Bottom left panel, miR-296-5p expression in MDA-MB-231 cells treated with increasing concentrations of 5-aza-2’-deoxycytidine (5-aza). Bottom central and right panels, quantification of CpG island methylation in MDA-MB-231 cells treated with 5-aza-2’-deoxycytidine (3μM; central, CpG-2; right, CpG-1). **(B)** miR-512-5p expression upon inhibition of DNA methylation as determined by quantitative TaqMan RT-PCR. Expression levels were normalized against RNU49. Top panel, Position of CpG islands analyzed for the miR-512-1 and miR-512-2 genes. Numbers indicate distance from miR-512-1. Bottom left panel, miR-512-5p expression in MDA-MB-231 cells treated with increasing concentrations of 5’-Aza-2’-deoxycytidine. Bottom central and right panels, quantification of CpG island methylation in MDA-MB-231 cells treated with 5-aza-2’-deoxycytidine (3μM; central, CpG-1; right, CpG-2). **(C)** miR-296-5p expression in TSA (1μM) or SAHA (5μM) treated MDA-MB-231 cells, as determined by quantitative TaqMan RT-PCR. Expression levels were normalized against RNU49. **(D)** miR-512-5p expression in TSA (1μM) or SAHA (5μM) treated MDA-MB-231 cells, as determined by quantitative TaqMan RT-PCR. Expression levels were normalized against RNU49. **(E)** miR-512-5p expression in MDA-MB-231 cells after treatment with the indicated chemical compounds, as determined by quantitative TaqMan RT-PCR. 5-aza-2’-deoxycytidine (1 μM) was added throughout the 5 days experimental period; TSA (1μM) was added at day 4; SAHA (5μM) was added at day 3. Expression levels were normalized against RNU49. **(F)** miR-296-5p expression in MDA-MB-231 cells after 4 days of treatment with the indicated inhibitors (TSA, 1μM; SAHA, 5μM; 5-aza-2’-deoxycytidine, 1 μM), as determined by quantitative TaqMan RT-PCR. Expression levels were normalized against RNU49. **(G)** hTERT expression in MDA-MB-231 cells after 4 days of treatment with the indicated inhibitors, as determined by quantitative RT-PCR. hTERT expression was normalized against actin. **(H, I)** Top panels, hTERT expression in MDA-MB-231 cells after 4 days of treatment with the indicated inhibitors (TSA 1μM; SAHA 5 μM; 5-aza-2’deoxycytidine 0.15 μM), as determined by quantitative RT-PCR. hTERT expression was normalized against actin. Bottom panels, western blotting analysis of MDA-MB-231 cells after the indicated treatments. n, number of independent experiments; error bars show standard deviation. P values of bisulfite sequencing were calculated using an unpaired student's t-test; P values of RT-PCR experiments were calculated using a Mann Whitney test; n.s., non significant - p-value >0,05.

Low miR-296-5p and miR-512-5p expression levels in basal type breast cancer cell lines suggest that the respective miR-296, miR-512-1 and miR-512-2 genes might be subjected to efficient repression or gene silencing. This prompted us to test whether epigenetic silencing of the miR-296-5p or miR-512-1 and miR-512-2 gene loci is aimed to ensure high hTERT expression. We treated MDA-MB-231 cells with DNA methyltransferase and histone deacetylase inhibitors and measured mature miR-512-5p and miR-296-5p expression levels by classic Taq-man PCR. DNA methylation analysis focused on CpG islands located in vicinity to the miRNA hosting genes, but also on CpG islands with a reported role in regulating miR-296-5p and miR-512-5p expression [37][42]. As expected, treatment with the DNMT inhibitor 5-aza-2'-deoxycytidine reduced DNA methylation levels of CpG rich regions of interest and mediated a 5 fold or 70 fold increase of miR-296-5p or miR-512-5p expression levels, respectively (Figure 5A, 5B). Treatment of MDA-MB-231 cells with the HDAC inhibitors trichostatin (TSA) or Vorinostat (suberoylanilide hydroxamic acid, SAHA) increased miR-296-5p expression levels 4 fold or 5 fold, respectively (Figure 5C). HDAC inhibitors did not significantly alter miR-512-5p levels, suggesting that inhibiting HDAC activity is not sufficient to override silencing of miR-512-5p expression by DNA methylation (Figure 5D). In line with this, combining 5-aza-2'-deoxycytidine with SAHA treatment caused a dramatic 90-fold increase of miR-512-5p levels compared to a 25-fold increase of miR-512-5p levels of MDA-MB-231 cells that where exclusively treated with 5-aza-2'-deoxycytidine (Figure 5E). Remarkably, TSA treatment does not affect miR-512-5p expression in this setup, indicating that the class I and II HDAC inhibitor SAHA has a specific relevance in regulating the epigenetic status of miR-512 genes. Similar to miR-512-5p, we show that a 5-fold increase of miR-296-5p in 5-aza-2'-deoxycytidine treated MDA-MD-231 cells increased to 32-fold or 42 fold when 5-aza-2'-deoxycytidine was used in combination with TSA or SAHA, respectively (Figure 5F). Real-time PCR analysis revealed that TSA or SAHA treatment does not significantly impact on the expression of HDACs, suggesting that observed alteration in miR-296-5p and miR-512-5p are not triggered via altered HDAC expression (Supplementary Figure 9A-9J). Remarkably, we found that treatment of MDA-MB-231 cells with SAHA and TSA reduced hTERT mRNA expression (Supplementary Figure 10A). SAHA and TSA were also able to reduce elevated hTERT expression of 5-aza-2'-deoxycytidine treated MDA-MB-231 cells. (Figure 5G). This is in line with previous studies that report that a treatment of cancer cells with HDAC inhibitors can reduce hTERT expression via an indirect epigenetic mechanism [46–50]. Of notice, the miR-296 and miR-512 loci respond to 5-aza-2’-deoxycytidine treatment already at lower concentration when compared to the hTERT gene (Supplementary Figure 10B). We consequently wished to test whether miR-296-5p and miR-512-5p are critical components that down-regulate hTERT expression in basal type breast cancer cells that are treated with epigenetic drugs. To achieve this, we antagonized TSA induced expression of miR-296 by transfecting MDA-MB-231 cells with antagomiR-296-5p and subsequently measured hTERT expression by RT-PCR. In this setup, transfection of antagomiR-296-5p significantly impaired TSA mediated reduction of hTERT expression (Figure 5H; top panel). In analogy to this, we found that competing increased miR-512-5p expression in SAHA and 5-aza-2’-deoxycytidine treated MDA-MD-231 cells by transfecting antagomiR-512-5p, rescued hTERT expression (Figure 5I; top panel). This demonstrates that epigenetic regulation of miR-296-5p and miR-512-5p impacts on hTERT expression levels in basal type breast cancer cells. Importantly, antagomiR-296-5p or antagomiR-512-5p transfection reduced apoptosis markers in drug treated MDA-MB-231 cells (Figure 5H, 5I; bottom panels). This highlights that DNA methylation and histone de-acetylation collaborate to silence miR-296-5p and miR-512-5p in basal type breast cancer cells to ensure increased hTERT expression, thus resulting in improved protection from apoptosis.

**Figure 6:**
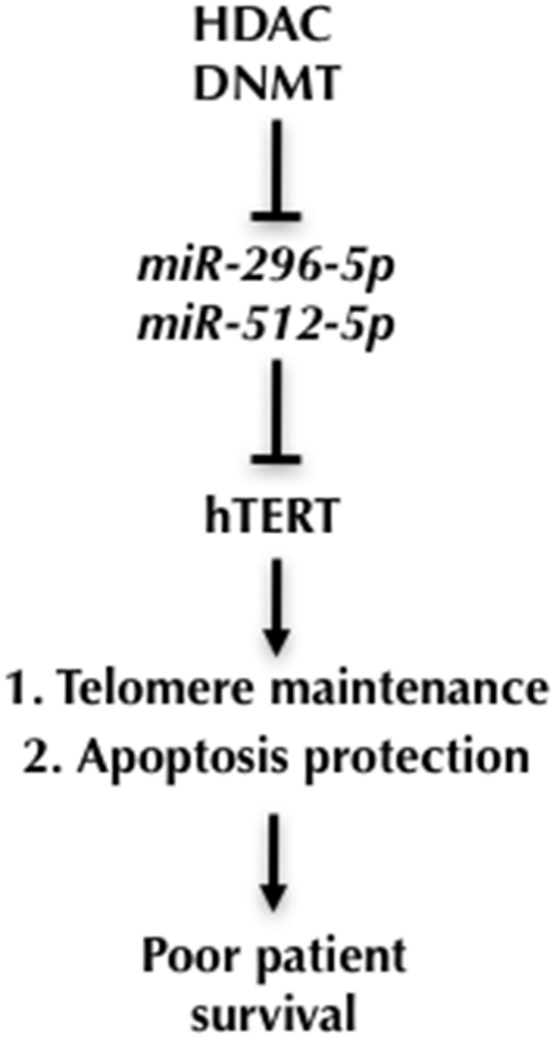
A model for miR-296-5p and miR-512-5p function in basal-type breast cancer Epigenetic silencing of miR-296 and miR-512 genes in basal type breast cancer cells releases hTERT from miRNA dependent suppression of gene expression. This leads to improved telomerase activity, enhanced telomere maintenance and protects from senescence and apoptosis, key factors that increase the aggressiveness of human breast cancer.

Altogether our data suggest a model where epigenetic silencing of miR-296-5p and miR-512-5p expression in basal breast cancer cells releases hTERT expression from miRNA mediated suppression of gene expression. This results in improved telomere maintenance and meliorates protection from cellular apoptosis. This pathway may contribute to the high biological aggressiveness of basal type breast cancer (Figure 6).

## DISCUSSION

The re-activation of telomerase expression allows the escape from replicative senescence and represents a key step during cancer formation [51]. In addition to its role on telomere maintenance, hTERT was demonstrated to promote cancer progression by suppressing apoptosis [10–16]. The expression of telomerase is subjected to direct control by central tumor suppressors and oncogenes underlining the tight link between hTERT and cancer formation [52]. Recent studies demonstrated that miR-138, miR-133a, miR-342, miR-491-5p, miR-541, miR-1207-5p, miR-1266 and miR-1182 control hTERT expression in leukemic T-cell lymphoblasts, gastric cancer, cervical cancer, thyroid carcinoma and head and neck squamous cell carcinoma [28–31, 33]. miR-512-5p was shown to target hTERT in head and neck squamous cell carcinoma [32]. To this end, miRNAs that control hTERT expression in breast cancer have not been discovered. Performing a high-throughput luciferase reporter assay we identified a panel of miRNAs that efficiently target the 3’UTR of hTERT. Out of this panel, miR-296-5p and miR-512-5p are significantly down-regulated in human breast cancer specimen. This is consistent with the requirement of telomerase expression in cancer cells and anticipates clinical relevance for miR-296-5p and miR-512-5p. miR-296-5p is encoded by the lincRNA Nespas that is transcribed from parental allele of the imprinted GNAS cluster located at chromosome 20q13.3 [53]. miR-296-5p has been shown to suppress cancer progression, metastasis, and neo-vascularization by targeting the expression of multiple genes including HMGA1, PUMA and SCRIB [35, 37, 54]. Mature miR-512-5p is encoded by the intergenic miR-512-1 and miR-512-2 genes located on human chromosome 19 and was reported to suppress apoptosis by targeting MCL-1 in gastric cancer and hTERT in head and neck squamous cell carcinoma [32, 43]. Functional validation revealed that miR-296-5p and miR-512-5p target specific sites in the 3’UTR of hTERT. Performing a series of gain and loss of function experiments we show that miR-296-5p and miR-512-5p have a relevant role in the direct regulation of hTERT expression in two different human breast cancer cell lines. However, given the complexity of hTERT gene expression control and the numerous mRNAs targeted by miR-296-5p and miR-512-5p, we cannot completely exclude that indirect effects contribute to miR-296-5p and miR-512-5p dependent control of hTERT expression. Importantly, due to dramatic differences of hTERT 3’UTR size and sequence content across vertebrate, targeting of hTERT by miR-296-5p and miR-512-5p is limited to human, chimpanzee and rhesus monkey. This suggests that miRNAs networks can control hTERT expression in a species-specific manner. Performing gain and loss of function experiments we found that hTERT expression and telomerase activity is under control of both, miR-296-5p and miR-512-5p. In line with this, we observed telomere shortening in cells overexpressing mature miR-296-5p or miR-512-5p. Of notice, recent studies report on telomere shortening in the context of reduced hTERT expression in cell model systems [55–59]. Several studies revealed an association of high telomerase expression with poor breast cancer prognosis [60–64]. The application of different types of patient treatment may have masked this correlation in a more recent study [65]. Here, we show that the link of high hTERT expression with poor survival is specific for the basal subtype of human breast cancer. Poor survival of basal type breast cancer with high hTERT expression appears to be recapitulated in specimen with enhanced expression of validated miR-296-5p or miR-512-5p target genes, including hTERT. This might provide first evidence that downregualtion of miR-296-5p, miR-512-5p in basal type breast cancer might help to promote the expression of a set of target genes, that could contribute to the aggressiveness of basal type breast cancer and hTERT expression in basal type breast cancer. Using a basal type breast cancer cell line, we show that increasing intracellular levels of mature miR-296-5p and miR-512-5p resulted in telomere shortening as well as induction of senescence and apoptosis, finally causing reduced cell proliferation. Rescue experiments in breast cancer cells expressing hTERT lacking the 3’UTR revealed that this proliferation defects can be directly attributed to miR-296-5p and miR-512-5p dependent down-regulation of hTERT. Together, this indicates that repression of miR-296-5p and miR-512-5p in basal type breast cancer cells releases hTERT from miRNA dependent repression, to enhance resistance to apoptosis and telomere maintenance, thus promoting cancer cell aggressiveness. These results are in line with the induction of apoptosis markers upon classic siRNA mediated depletion of hTERT [10, 11]. Recently, miR-512-5p has been shown to modulate the expression of the apoptosis regulator MCL-1, and miR-296-5p has been demonstrated to reduce cell proliferation of breast and prostate cancer cells by targeting SCRIB or HMGA1, respectively [35, 36, 42]. This suggests that miR-512-5p and miR-296-5p act as potent tumor suppressive miRNAs that exert their anti-proliferative function along multiple pathways that include the regulation of hTERT, the catalytic subunit of the telomerase complex. Low expression of miR-296-5p and miR-512-5p in breast cancer indicates that miR-296 and miR-512 hosting gene expression is tightly regulated. DNA methylation at Alu-repeats in vicinity to miR-512 genes suggested the involvement of epigenetic regulatory mechanisms [43]. Antagonizing DNA methylation using 5-aza-2'-deoxycytidine caused an efficient up-regulation of mature miR-296-5p and miR-512-5p in MDA-MB-231 cells that was enhanced when 5-aza-2'-deoxycytidine was combined with HDAC inhibitors: miR-296-5 pexpression was increased upon TSA treatment, a reported inhibitor of class I and II HDACs; in contrast, miR-512-5p was increased upon treatment with the pan-class I and II HDAC inhibitor Vorinostat (SAHA). This suggests that different HDACs collaborate with DNMTs to ensure efficient epigenetic silencing of miR-296 or miR-512. We show that loss of DNA methylation increases hTERT expression on the RNA level, whereas inhibition of HDACs using TSA or SAHA resulted in a reduction of hTERT mRNA levels. This suggests the existence of an indirect mechanism, triggered by HDAC inhibitors that impinges on hTERT expression. Importantly, hTERT expression was restored in TSA/SAHA treated cells upon transfection with antagomiR-296-5p or antagomiR-512-5p. This suggests that TSA/SAHA treatment increases miR-296-5p and miR-512-5p levels, resulting in reduced hTERT expression. Consistent with elevated hTERT expression, antagomiR treated cells displayed increased resistance to apoptotic stimuli induced by HDAC inhibitor treatment. This is in line with a role for hTERT in protecting from apoptosis. Together, this demonstrates that miR-296-5p and miR-512-5p have a central role in executing epigenetic regulatory circuits that control hTERT expression and telomere length in cancer cells. Our work identifies two important tumor-suppressor miRNAs that control the expression of the catalytic component of telomerase. In particular, our data suggest that DNA methylation and histone de-acetylation collaborate as redundant epigenetic mechanisms to silence the expression of miR-296-5p and miR-512-5p in basal type breast cancer cells. This, in turn, leads to elevated expression of hTERT that promotes cancer aggressiveness by improving telomere maintenance and resistance to apoptosis and senescence (Figure 6). The observation of reduced distant metastasis free survival and relapse free survival of basal type breast cancer patients with increased miR-296-5p and miR-512-5p target gene expression signatures suggest that epigenetic regulation of miR-296 and miR-512 may be of relevance in this breast cancer subtype. Future efforts should aim to use a large set of specimen obtained from basal type breast cancer in order to experimentally validate the link between epigenetic silencing of miR-296 and miR-512, increased expression of validated miR-296-5p/miR-512-5p target genes and telomere homeostasis in a clinical setting. A better understanding of biological mechanism underlying the aggressive biology of basal type breast cancer is key for a better treatment for this subtype of breast cancer. The interplay between epigenetic gene silencing and miR-296/miR-512 to favor hTERT expression identifies basal type breast cancer as potential breast cancer subtype to explore telomerase related therapeutic approaches. Along these lines, the exploration of epigenetic inhibitors in more complex preclinical models could provide valuable insights into the relevance of miR-296 and miR-512 in imposing an anti-tumor effect that includes telomere related pathways in basal type breast cancer.

## MATERIALS AND METHODS

### Cell lines and culture

Cell lines used were obtained from ATCC and have not been cultured for longer than 6 months. MCF-7 (Michigan Cancer Foundation-7, breast adenocarcinoma), SK-BR-3 (breast adenocarcinoma derived) and T-47D (breast ductal carcinoma) cells were cultured in *RPM*I 1640, 10% heat inactivated fetal bovine serum (FBS, Gibco). Instead, MDA-MB-231 (breast adenocarcinoma), MDA-MB-468 (breast adenocarcinoma) and MDA-MB-157 (mammary gland, medullary carcinoma) and HeLa (cervix adenocarcinoma) cells were cultured in DMEM with 10% heat inactivated FBS. MDA-MB-231 cells transduced with retroviral miRNA-minigene or shTERT vectors were selected with blasticidin (8μg/mL) or puromycin (2μg/mL), respectively. MDA-MB-231 cells infected with pBabe empty were selected with hygromycin (100μg/mL). MDA-MB-231 cells were treated with 5-aza-2’-deoxycytidine (Sigma), Trichostatin A (Sigma) or Suberoylanilide hydroxamic acid (SAHA) (Sigma) at concentrations indicated in respective figure legends.

### FACS analysis

Cell cycle FACS analysis: after indicated treatments, cells were washed in 1X PBS and fixed with ice-cold ethanol for 30 minutes at -20 °C. Subsequently, cells were washed with 1X PBS and re-suspended in 1x PBS with 0,1% NP40 and 2μl of RNase A (10mg/ml) and incubated for 10 minutes at RT. Propidium iodide (Sigma) was added to a final concentration of 50 μg/ml and cells were subjected to flow cytometry. Annexin V FACS analysis: apoptosis rate was measured using Dead Cell Apoptosis Kit with Annexin V Alexa Fluor488 & Propidium Iodide (PI) (Invitrogen V13241), according to the manufacturer's suggestions. For flow cytometry, a FACSCalibur flow cytometer (BD Biosciences) operated by CellQuest software was used; at least 30,000 events were collected per sample. Results were analyzed using the FlowJo software (Tree Star, Ashland, OR, USA).

### β-galactosidase assay

After repeated mimic-miRNA siRNA transfection (twice during 6 days) cells were washed in 1xPBS and fixed in 2% formaldehyde/0.2% glutaraldehyde for 15 minutes at room temperature. Cells treated with 100 nM Adriamycin were used as positive control. Cells were washed and incubated with fresh SA-β-gal staining solution containing 1mg/ml X-gal, 40 mM citric acid/sodium phosphate (pH 6.0), 5 mM potassium ferrocyanide, 5 mM potassium ferrocyanide, 150 mM NaCl and 2 mM MgCl2 for 16-18 h at 37 °C. Blue-stained senescent cells were counted using a light microscopy (Olympus IX71, Olympus America, PA, USA); images were captured using a digital camera (Olympus-Camedia C-5060).

### Western blotting

Whole cell lysates were prepared using a modified RIPA buffer (20 mmol/L Tris-HCl (pH 7.5) 350 mmol/L NaCl, 1 mmol/L Na2EDTA, 1 mmol/L EGTA, 1% NP-40, 1% sodium deoxycholate, 2.5 mmol/L sodium pyrophosphate, 1 mmol/L β-glycerophosphate, 1 mmol/L Na3VO4, 1 mg/mL leupeptin). Samples were supplemented with complete protease inhibitor (Roche) and were sonicated followed by centrifugation. Supernatants were recovered and used for Western blotting according to standard procedures. Primary antibodies: mouse anti-actin (clone AC-74, Sigma, A2228), mouse anti-γH2AX (ser139) (JBW301, Millipore, 06-536), rabbit anti-p-p53 (ser15) (Cell Signalling, 9284), rabbit anti-Cleaved caspase-3 (Asp 175) (Cell Signalling, 9661), mouse anti-PARP-1 (Calbiochem, AM30), rabbit anti-PARP (Cell Signaling, 9542), rabbit anti-p21 (Cell Signaling, 2947), rabbit anti-p21 (C-19, Santa Cruz, 397), mouse anti-α-tubulin (Calbiochem, DM1A), rabbit anti-Caspase-3 (Cell Signaling, 9662). After extensive washing primary antibodies were incubated with the specific secondary antibodies bound to the HRP enzyme (horseradish peroxidase-conjugated antibody) (GE Healthcare) and subjected to chemiluminescence using the ECL system (GE Healthcare).

### Luciferase reporter assays

Computational target prediction analysis using PITA, TargetScan and the micro-RNA.org targets and expression tool identified miRNAs with *in-silico* target specificity for the 3’UTR of hTERT [26]. For luciferase reporter assays, the hTERT 3’UTR was cloned downstream of Renilla luciferase (psiCHECK2 vector, Promega). In addition, psiCHECK2 contains a Firefly luciferase cassette that allows to control for transfection efficiency. Transcription from the SV40 early promoter/enhancer generates a transcript containing the Renilla luciferase coding sequence, fused to the 3’UTR of hTERT. High-throughput luciferase reporter assay: HeLa cells were co-transfected in a 48 well-plate with 18ng of hTERT 3’UTR luciferase reporter plasmid and candidate mimic-miRNA siRNAs (final concentration: 50nM). 72 hours post-transfection, cells were lysed in LBL lysis Buffer (Promega) and Renilla/Firefly luciferase reporter activity was measured using a Dual-Luciferase Reporter Assay System (Promega) and a GloMax® 96 Microplate Luminometer (Promega). Mutant TERT-3’-UTR reporter constructs were generated using the QuickChange II XL Site-Directed Mutagenesis kit according to the manufacturer's suggestions (Agilent): TERT-Δ479-481 Fw: CCTGCACCTGGAGAAGGGTCCCTGTGGGTCAAATTG; TERT-Δ479-481 Rev: CAATTTGACCCACAGGGACCCTTCTCCAGGTGCAGG; TERT-Δ83-85 FW: AGGGAGGGAGGGGCAAACCACACCCAGGCCC; TERT-Δ83-85 Rev: GGGCCTGGGTGTGGTTTGCCCCTCCCTCCCT; TERT-Δ164-166 FW: CCTGCATGTCCGGCTGAAGGCTTGTTGTCCGGCTGAG; TERT-Δ164-166 Rev: CTCAGCCGGACAACAAGCCTTCAGCCGGACATGCAGG. Constructs were sequence-verified.

### Telomere repeat amplification protocol (TRAP assay)

Telomerase activity was measured in MCF-7 and MDA-MB-231 cells, using the TRAPeze Telomerase Detection Kit (Millipore S7700) [66]. Cells were transiently transfected with indicated siRNA or RNA-oligonucleotides. Six days post-transfection, cells were washed with 1X PBS and lysed with 1X CHAPS buffer for 30 minutes on ice. Samples were centrifuged at 12,000 g for 20 minute at 4 °C. For TRAP, 125 and 50 ng of the supernatant fraction was incubated at 30 °C for 30 minutes and subjected to a PCR, using DreamTaq DNA Polymerase (Thermo Scientific). Heat-inactivated lysates and TSR8 were used as control. Samples were then loaded in a 10% non-denaturing polyacrylamide gel in 0,5 X TBE buffer and run for 1,5 hours at 400 volts. Subsequently, the gel was stained with SYBR Gold nucleic acid gel (Life Technologies) according to the manufacturer's suggestion; a gel DOC XR system (BIO-RAD) was used for the acquisition of images.

ImageJ Software was used to determine the intensity of the TRAP products. Non-heat-treated samples, (x) and heat-treated samples, (x0); primer-dimer/PCR contamination control, (r0) and positive PCR control - TSR8 (r). The signal from S-IC (Standard internal PCR control) in non-heat-treated samples (c) and TSR8 control (cr) was measured. The following formula was used to calculate TRAP activity: (x−x0)/c(r−r0)/cr x100.

### Telomere DNA FISH

Interphase Telomere DNA FISH was performed as previously described [67]. Captured images were analyzed using a Zeiss Axiovert 200M microscope, equipped with an AxioCam MRm Zeiss digital camera and the AxioVision Rel. 4.8 imaging software. For quantitative DNA telomere FISH analysis at least 40 nuclei (for each experimental condition) were determined using spot IOD analysis using the TFL-TELO Software. Student's t-test was used to calculate statistical significance. To provide better overview on telomere length alterations, telomere length distribution was split into a long (>10.000 a.f.u.), medium (5.000-10.000 a.f.u.) and short telomere (<5.000 a.f.u.) group.

### Bisulfite sequencing

MDA-MB-231 cells were treated with 5-Aza-2′-deoxycytidine (3 μM; Sigma-Aldrich) for 5 days. Cells were then re-suspended and digested in protein K buffer (Ambion). Genomic DNA was purified by phenol/chloroform/isoamyl alcohol (Fisher scientific) extraction. A total of 500 ng of genomic DNA was subjected to bisulfite conversion using the Qiagen EpiTect Bisulfite kit (Qiagen) following the manufacturer's instructions. Respective CpG islands were PCR amplified using primer sets that were designed using MethPrimer software [68]. miR-296 CpG-1 island (1751bp upstream of miR-296): Fw: 5’-GTGAAAGTAAGTTTTATTGATGGT-3’; Rv_ 5’-CAAAAAATTCCAAAAACCCTTAAA-3’, [37]; miR-296 CpG-2 island (88bp downstream of miR-296) : Fw: 5’-GTGTTAGGAGTGGAGATAGGATAGT-3’; Rv: 5’-TCAATAAAAATAAAAAAAACCTCC-3’; miR-512- CpG-1 island (2806bp upstream of miR-512-1): Fw: 5’-TTGTAATTTTAGTATTTTGGGAGGT-3’; Rv: 5’-AAAACAATCTCACTCTATTACCCAAAC-3’; miR-512 CpG-2 island, regulating the C19MC cluster (17692 bp upstream of miR-512-1) Fw_5’-TTTTTTTTGAGGGATTAGAATTTGTT-3’ RV_5’-CCCTAAACTTCCTAATTAAATAAAAAACTA-3’ PCR products were gel purified and cloned into pCR2.1 using the TA cloning kit (Invitrogen). Individual clones were sequenced with M13 Fw and M13 Rev primers. Obtained sequences were analyzed using QUMA software.

### Bioinformatics on clinical data from breast cancer patients

miRNA expression values in breast cancer samples were obtained from EMBL-EBI EGAS019575R200122 [34]. Gene expression values, clinical data and survival data were obtained using KM plotter and GOBO [44, 45]. To evaluate the impact of gene signatures on breast cancer survival the patient samples were split into two groups according to various quantile expressions (median for GOBO and using the best cut-off algorithm or KM plotter) of the proposed signatures, a Mantel-Haenszel test was applied and was obtained a Kaplan–Meier survival curve. Statistical analysis has been performed using R software environment for statistical computing (R Core Team (2015). R: A language and environment for statistical computing. R Foundation for Statistical Computing, Vienna, Austria. http://www.R-project.org/) and Bioconductor [69].

## SUPPLEMENTARY MATERIALS FIGURES


