# Supplementary material for: Epigenetic silencing of miR-296 and miR-512 ensures hTERT dependent apoptosis protection and telomere maintenance in basal-type breast cancer cells
Source: Oncotarget. 2017 Sep 23;8(56):95674–91. doi: 10.18632/oncotarget.21180 (PMC5707052; doi:10.18632/oncotarget.21180)
Supplement: Supplementary file 1 [file oncotarget-08-95674-s001.pdf]

Epigenetic silencing of miR-296 and miR-512 ensures hTERT dependent apoptosis protection and telomere maintenance in basal-type breast cancer cells

SUPPLEMENTARY MATERIALS

Plasmids

p-Babe-hygro-hTERT pBabe-hTERT and pMKO.1-puro hTERT shRNA were purchased from Addgene (plasmids 1773 and 10688). miR-296 vector was obtained from a published miRNA-expression vector library [7]. A miR-512 mini-gene vector was generated by amplifying the genomic region if miR-512 using GC-GGATCCCCAAAGTGCTGGGATTACAG; GCGAATTC- AAGAGGCAACCAATCCAGAC. PCR products were cloned into pMESV [7]. hTERT luciferase reporter vectors were generated by PCR amplication of the hTERT 3'UTR using the following oligonucleotides: TERT-3'-UTR-Fw: GCCGCGGC CGCTGGCCACCCGCCCACAGCCAG, TERT-3'-UTR-Rv: GCCGCGGCCGCCAAAACTGAAAAAC TCATATATTCAG. NotI digested PCR products were cloned into psiCHECK2 (Promega).

Transfection of siRNA and RNA-oligonucleotides

Cells were transfected with ON-TARGETplus smartpool hTERT siRNAs (Dharmacon), ON-TARGETplus non-targeting siRNA#1 (Dharmacon), hsa-miR-296-5p or has-miR-512-5p mimic siRNAs (Dharmacon), miRIDIAN microRNA hsa-miR-296-5p or hsa-miR-512-5p haripin inhibitor (Dharmacon) or miRIDIAN microRNA mimic negative control siRNA (Dharmacon), using RNAiMAX Lipofectamine (Invitrogen) at a final concentration of 30nM according to the manufacturer's suggestions.

Quantitative RT-PCR

Total RNA was prepared using TRIzol reagent (Gibco/BRL) according to the manufacturer's protocol. For miRNA analysis 10 ng of RNA were reverse transcribed using the hsa-miR-296-5p, hsa-miR-512-5p and RNU49 TaqMan™ MicroRNA Assay systems (Applied Biosystems). The stem loop real-time PCR was performed by using Taqman Fast PCR Master mix (Life Technologies), according to the manufacturer's suggestions. Quantitative miRNA expression data was analyzed using a Bio-Rad CFX96 C1000 Touch Real-time PCR Detection System. For quantitative mRNA expression analysis 500 ng of total RNA were reverse transcripted using the QuantiTect Reverse Transcription kit (QIAGEN) according to the manufacturer's suggestions. Quantitative PCR was performed using the SYBR Green Master Mix (Applied Biosystem) and analyzed with a StepOnePlus real time PCR machine (Applied Biosystem). mRNA levels were normalized to actin. PCR primers used for quantitative real-time PCR: hTERT_FW: 5'-AACAAGCTGTTTGCGGGGAT-3'; hTERT_REV: 5'-CCAGGGTCCTGAGGAAGGTTT-3'; β-ACTIN_FW: 5'-AGCACTGTGTTGGCGTACAG-3'; β-ACTIN_RV: 5'-TCCCTGGAGAAGAGCTACGA-3'; HDAC1_FW 5'-AGAATGCTGCCGCACGCACC-3'; HDAC1_RV 5'-CGGGGCCTTGGTTTCTGTCCC-3'; HDAC2_FW 5'-CTTCCCCGCGGGACTATCGC-3'; HDAC2_RV 5'-TCGGCAGTGGCTTTATGGGGC-3'; HDAC3_FW 5'-CCTGGCATTGACCCATAGCC-3'; HDAC3_RV 5'-CTCTTGGTGAAGCCTTGCATA-3'; HDAC4_FW 5'-AATCTGAACCACTGCATTTCCA-3'; HDAC4_RV 5'-GGTGGTTATAGGAGGTCGACACT-3'; HDAC5_FW 5'-TTGGAGACGTGGAGTACCTTACAG-3'; HDAC5_RV 5'-GACTAGGACCACATCAGGTGAGAAC-3'; HDAC6_FW 5'-TGGCTATTGCATGTTCAACCA-3'; HDAC6_RV 5'-GTCGAAGGTGAACTGTGTTCCT-3'; HDAC7_FW 5'-CTGCATTGGAGGAATGAAGCT-3'; HDAC7_RV 5'-CTGGCACAGCGGATGTTTG-3'; HDAC8_FW 5'-TCCCGAGTATGTCAGTATATATGA-3'; HDAC8_RV 5'-GCTTCAATCAAAGAATGCACCAT-3'; HDAC9_FW 5'-GAATCCTCAGTCAGTAGCAGTTC-3'; HDAC9_RV 5'-GGGGCAAAACCGAAGTCTCAT-3'; HDAC10_FW 5'-TGGGAAGCTCCTGTACCTCTT-3'; HDAC10_RV 5'-GGCTGGAGTGGCTGCTATAC-3'; MN1_FW 5'-TGTGTTCTTTGAGAGGTTCAGTG-3'; MN1_RV 5'-GCTGCATTAACGGGTGCCT-3'; DBF4_FW 5'-ATGAACTCCGGAGCCATGAG-3'; DBF4_RV 5'-CTCGCCCTCCCAGATCCTTA-3'; LZTR1_FW 5'-GTGCTGGTTGTGTGCGAGAG-3'; LZTR1_RV 5'-GCAGTGCTCCTTGAGTTGGC-3'; MAPKAPK3_FW 5'-AGATAATGCGGGATATTGGCAC-3'; MAPKAPK3_RV 5'-TGTGTAGAGTAGGTTTTCAGGCT-3';

COPZ1_FW 5'-GATGGAGATCGACTTTTTGCCA-3'; COPZ1_RV 5'-TCAGTCCGATGGGTCTTGTTG-3' ; MCL1_FW 5'-ATGCTTCGGAAACTGGACAT-3'; MCL1_RV 5'-TCCTGATGCCACCTTCTAGG-3' CD44_FW 5'-GGTCCTATAAGGACACCCCAAAT-3'; CD44_RV 5'-AATCAAAGCCAAGGCCAAGA-3'; IKBKE_FW 5'-ACTCTGGAAGTGGCAAGGACAT-3'; IKBKE_RV 5'-TACCTGATCCCGGCTCTTCACCA-3'; WNK4_FW 5'-GTGAAGGCTGCGGAAGACTC-3'; WNK4_RV 5'-CTGGGTCTCCATGTCCTCCTT-3' ; HGS_FW 5'-CTCCTGTTGGAGACAGATTGGG-3'; HGS_RV 5'-GTGTGGGTTCTTGTCGTTGAC-3'; PUMA_FW 5'-ATGGCGGACGACCTCAAC-3'; PUMA_RV 5'-AGTCCCATGAAGAGATTGTACATG AC-3'

Statistical analysis

Unless otherwise indicated in figure legends, statistical analysis of experiments with 3-5 replicas was performed using a non-parametric Man Whitney test. In figures with replica numbers >5 an unpaired students t-test was used. Information on statistical methods for analysis of patient data is mentioned in the respective figure legends.

SUPPLEMENTARY REFERENCES

1. Dvinge H, Git A, Gräf S, Salmon-Divon M, Curtis C, Sottoriva A, Zhao Y, Hirst M, Armisen J, Miska EA, Chin SF, Provenzano E, Turashvili G, et al. The shaping and functional consequences of the microRNA landscape in breast cancer. Nature. 2013; 497: 378-82.
2. Masutomi K, Possemato R, Wong JM, Currier JL, Tothova Z, Manola JB, Ganesan S, Lansdorp PM, Collins K, Hahn WC. The telomerase reverse transcriptase regulates chromatin state and DNA damage responses. Proc Natl Acad Sci USA. 2005; 102: 8222-7.
3. Ringnér M, Fredlund E, Häkkinen J, Borg Å, Staaf J. GOBO: gene expression-based outcome for breast cancer online. PLoS One. 2011; 6: e17911.
4. Dinami R, Ercolani C, Petti E, Piazza S, Ciana Y, Sacconi A, Biagioni F, le Sage C, Agami R, Benetti R, Mottolese M, Schneider C, et al. miR-155 drives telomere fragility in human breast cancer by targeting TRF1. Cancer Res. 2014; 74: 4145-56.
5. Hu Z, Fan C, Oh DS, Marron JS, He X, Qaqish BF, Livasy C, Carey LA, Reynolds E, Dressler L, Nobel A, Parker J, Ewend MG, et al. The molecular portraits of breast tumors are conserved across microarray platforms. BMC Genomics. 2006; 7: 96.
6. Bernard PS, Parker JS, Mullins M, Cheung MC, Leung S, Voduc D, Vickery T, Davies S, Fauron C, He X, Hu Z, Quackenbush JF, Stijleman IJ, et al. Supervised risk predictor of breast cancer based on intrinsic subtypes. J Clin Oncol. 2009; 27: 1160-7.
7. Voorhoeve PM, le Sage C, Schrier M, Gillis AJ, Stoop H, Nagel R, Liu YP, van Duijse J, Drost J, Griekspoor A, Zlotorynski E, Yabuta N, De Vita G, et al. A genetic screen implicates miRNA-372 and miRNA-373 as oncogenes in testicular germ cell tumors. Cell. 2006; 124: 1169-81.
8. Wei JJ, Wu X, Peng Y, Shi G, Basturk O, Olca B, Yang X, Daniels G, Osman I, Ouyang J, Hernando E, Pellicer A, Rhim JS, et al. Regulation of HMGA1 expression by microRNA-296 affects prostate cancer growth and invasion. Clin Cancer Res. 2011; 17: 1297-305.
9. Vaira V, Faversani A, Dohi T, Montorsi M, Augello C, Gatti S, Coggi G, Altieri DC, Bosari S. miR-296 regulation of a cell polarity-cell plasticity module controls tumor progression. Oncogene. 2012; 31: 27-38.
10. Cazanave SC, Mott JL, Elmi NA, Bronk SF, Masuoka HC, Charlton MR, Gores GJ. A role for miR-296 in the regulation of lipoapoptosis by targeting PUMA. J Lipid Res. 2011; 52: 1517-25.
11. Robson JE, Eaton SA, Underhill P, Williams D, Peters J. MicroRNAs 296 and 298 are imprinted and part of the GNAS/Gnas cluster and miR-296 targets IKBKE and Tmed9. RNA. 2012; 18: 135-44.
12. Mao J, Li C, Zhang Y, Li Y. Human with-no-lysine kinase-4 3'-UTR acting as the enhancer and being targeted by miR-296. Int J Biochem Cell Biol. 2010; 42: 1536-43.
13. Würdinger T, Tannous BA, Saydam O, Skog J, Grau S, Soutschek J, Weissleder R, Breakefield XO, Krichevsky AM. miR-296 regulates growth factor receptor overexpression in angiogenic endothelial cells. Cancer Cell. 2008; 14: 382-93.
14. Li J, Lei H, Xu Y, Tao ZZ. miR-512-5p suppresses tumor growth by targeting hTERT in telomerase positive head and neck squamous cell carcinoma *in vitro* and *in vivo*. PLoS One. 2015; 10: e0135265.
15. Saito Y, Suzuki H, Tsugawa H, Nakagawa I, Matsuzaki J, Kanai Y, Hibi T. Chromatin remodeling at Alu repeats by epigenetic treatment activates silenced microRNA-512-5p with downregulation of Mcl-1 in human gastric cancer cells. Oncogene. 2009; 28: 2738-44.

A.

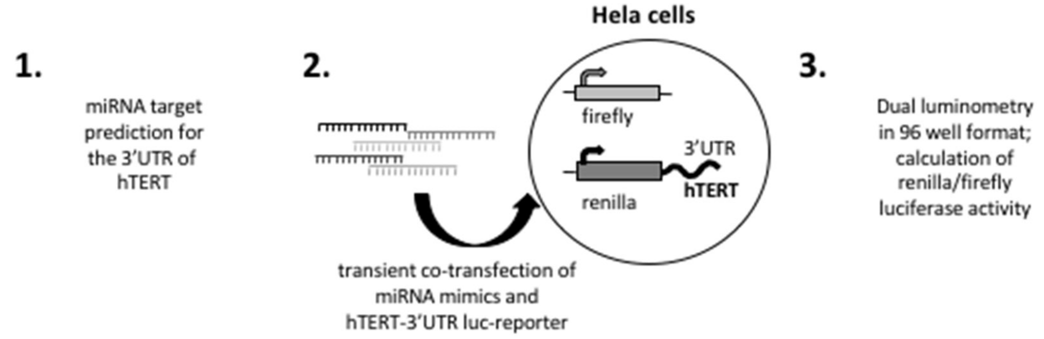

B.

	Experiment 1		Experiment 2				
miRNA	Rank	renilla/firefly ratio	Rank	renilla/firefly ratio	average renilla/firefly ratio	SD	average ranking
hsa-miR-541	1	0,16	1	0,16	0,16	0,00	1,0
hsa-miR-637	2	0,36	2	0,19	0,27	0,12	2,0
hsa-miR-1207-5p	9	0,55	4	0,30	0,43	0,18	6,5
hsa-miR-16-1-3p	3	0,37	7	0,53	0,45	0,11	5,0
hsa-miR-661	4	0,39	8	0,57	0,48	0,13	6,0
hsa-miR-608	6	0,44	9	0,57	0,50	0,09	7,5
hsa-miR-296-5p	7	0,45	10	0,58	0,51	0,09	8,5
hsa-miR-512-5p	11	0,66	6	0,42	0,54	0,17	8,5
hsa-miR-1234	5	0,42	11	0,70	0,56	0,20	8,0
hsa-miR-491-5p	21	1,00	3	0,25	0,62	0,53	12,0
hsa-miR-296-3p	10	0,56	18	1,05	0,81	0,34	14,0
hsa-miR-423-5p	12	0,75	15	0,94	0,84	0,13	13,5
hsa-miR-299-3p	13	0,82	13	0,89	0,85	0,05	13,0
hsa-miR-532-3p	16	0,88	12	0,83	0,86	0,04	14,0
hsa-miR-1323	8	0,51	23	1,26	0,88	0,53	15,5
hsa-miR-1275	26	1,43	5	0,35	0,89	0,76	15,5
hsa-miR-138	14	0,85	16	1,00	0,93	0,11	15,0
hsa-miR-760	19	0,99	14	0,90	0,95	0,06	16,5
hsa-miR-663	17	0,90	17	1,01	0,96	0,08	17,0
hsa-miR-548a-3p	15	0,87	19	1,11	0,99	0,17	17,0
hsa-miR-548f	18	0,93	20	1,12	1,02	0,14	19,0
hsa-miR-1249	23	1,18	22	1,24	1,21	0,04	22,5
hsa-miR-548e	25	1,37	21	1,16	1,26	0,15	23,0
hsa-miR-1182	20	0,99	25	1,57	1,28	0,41	22,5
hsa-miR-1266	22	1,17	24	1,48	1,32	0,22	23,0
hsa-miR-548o	24	1,35	26	1,66	1,51	0,22	25,0
hsa-miR-1307	28	2,61	28	1,98	2,29	0,44	28,0
hsa-miR-632	27	2,56	29	2,14	2,35	0,30	28,0
hsa-miR-1268	29	3,29	27	1,98	2,63	0,92	28,0

italic: miRNAs previously reported to target hTERT

Supplementary Figure 1: **(A)** Strategy of the luciferase reporter assay to identify miRNAs that target the 3'UTR of hTERT. **(B)** Results of the high-throughput hTERT luciferase reporter assay. Experiments were carried out in duplicate. Renilla to firefly ratios <1 indicate target specificity for individual candidate miRNAs for the hTERT 3'UTR.

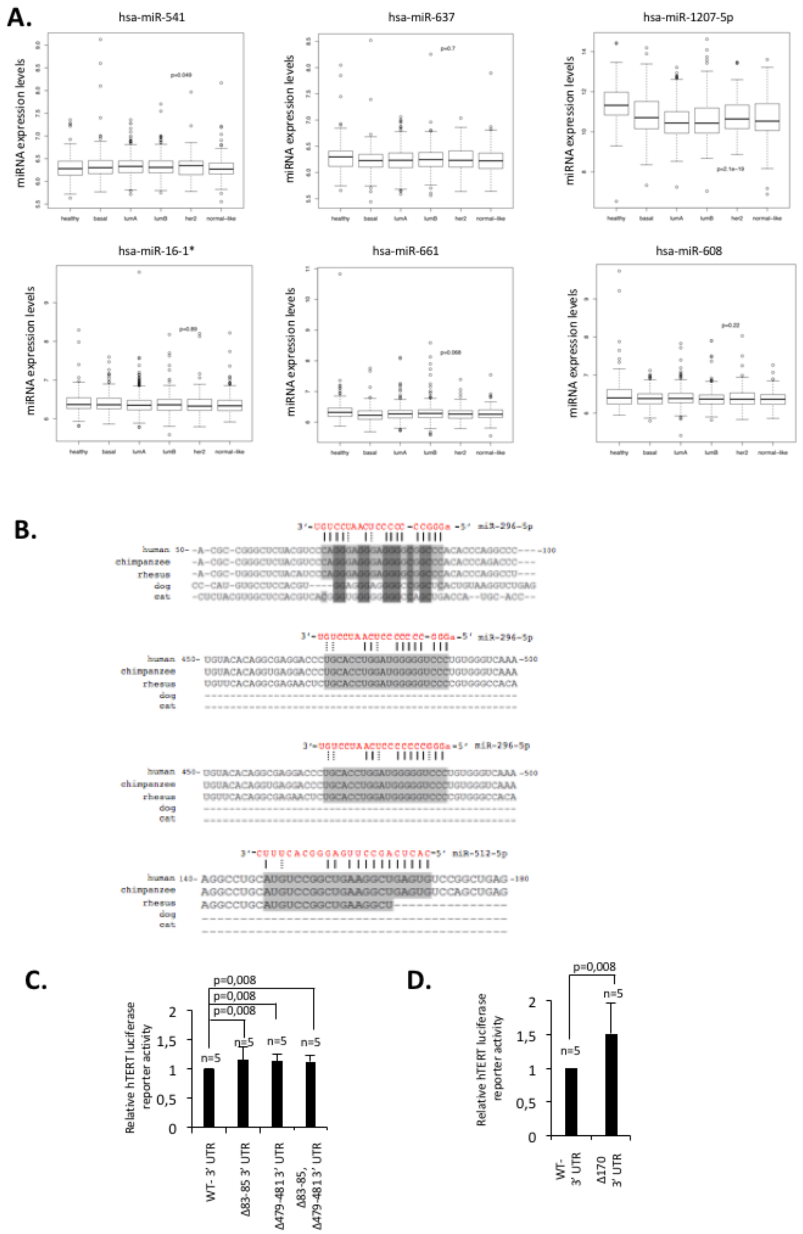

Supplementary Figure 2: (A) Box blots show expression levels of candidate miRNAs in normal breast tissue and human breast cancer subtypes using the METABRIC-dataset [1]. Expression values are shown in box blots at a log2 scale; box extremes indicate first and third quartile, whiskers extend to the extreme values included in the interval calculated as +/-1.58 IQR/sqrt(n) where IQR (interquartile range) is calculated as the third quartile minus the first quartile; p-values are calculated comparing normal breast against all cancer samples expression levels (Wilcox test). **(B)** Sequence alignment of miR-296-5p and miR-512-5p to the hTERT 3'UTR of the indicated species. **(C, D)** Luciferase reporter assays using Hela cells transfected with wild-type hTERT 3'UTR luciferase reporter or the indicated hTERT 3'UTR luciferase reporters carrying mutations for miR-296-5p target sites (C) or miR-512-5p target sites (D). Mutations in the hTERT 3'UTR increase luciferase reporter activity. n, number of independent experiments; error bars show standard deviation, E-M.: p values were calculated using a Mann Whitney test.

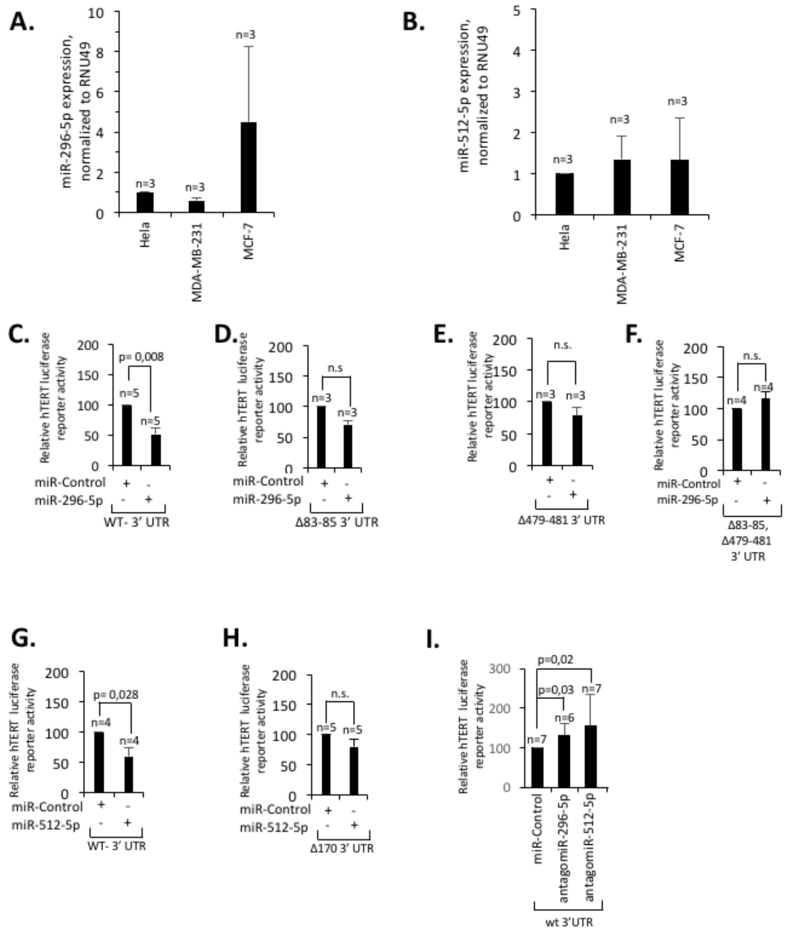

Supplementary Figure 3: (A, B) miR-296-5p (A) and miR-512-5p (B) expression in HMEC, Hela, basal type MDA-MB-231 and luminal type MCF7 cells, as determined by quantitative TaqMan RT-PCR. miR-296-5p and miR512-5p levels were quantified against RNU49. Expression of HMEC was set "1" **(C-F)** Luciferase reporter assays in Hela cells using a wild-type hTERT 3'UTR (C) or hTERT 3'UTR constructs that contain mutations of the respective miR-296-5p target sites (D-F). Mutations of miR-296-5p target sites result in increased luciferase reporter activity, when compared to control miRNA transfected cells. **(G-H)** Luciferase reporter assays in Hela cells using a wild-type hTERT 3'UTR (G) or a hTERT 3'UTR construct that contains a mutation of the miR-512-5p target site (H). Mutations of miR-512-5p target sites result in increased luciferase reporter activity, when compared to control miRNA transfected cells. **(I)** hTERT 3'UTR luciferase reporter assay after transient transfection of the indicated miRNA inhibitors in MDA-MB-231 cells. n, number of independent experiments; error bars show standard deviation, p values were calculated using a Mann Whitney test; n.s., non significant - p-value >0,05.

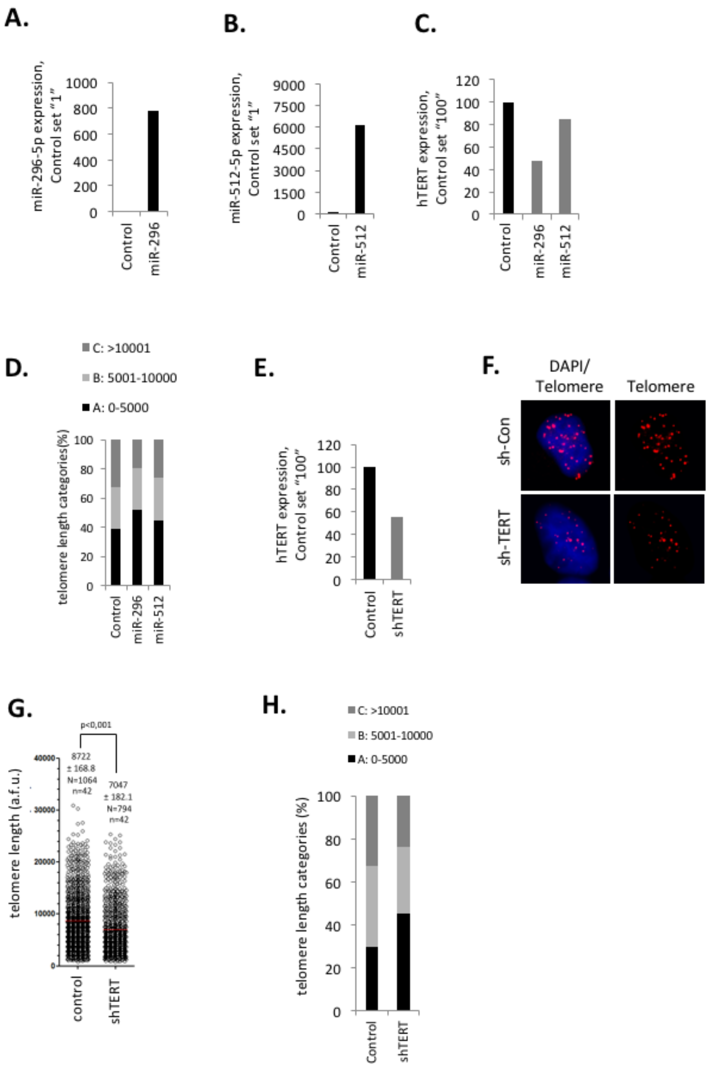

Supplementary Figure 4: (A) miR-296-5p expression in MDA-MB-231 transduced with a retroviral vector containing a miR-296 mini-gene, as determined by quantitative TaqMan RT-PCR; Expression values were quantified against RNU49. **(B)** miR-512-5p expression in MDA-MB-231 transduced with a retroviral vector containing a miR-512 mini-gene, as determined by quantitative TaqMan RT-PCR; Expression values were quantified against RNU49. **(C)** hTERT expression in MDA-MB-231 transduced with a retroviral vector containing a miR-296 or a miR-512 mini-gene, as determined by quantitative RT-PCR; Expression values were quantified against actin. **(D)** Ectopic miR-296-5p and miR-512-5p cause an increase in the short-telomere fraction (a.u.f. <5.000) and a decrease of the long-telomere fraction (a.u.f. >10.000. **(E)** hTERT expression in MDA-MB-231 transduced with a retroviral vector containing a shTERT construct [2], as determined by quantitative RT-PCR; Expression values were quantified against actin. **(F)** Representative images of telomere DNA-FISH on MDA-MB-231 cells transduced with retroviral vectors encoding an shTERT construct. Genomic DNA was stained using DAPI. **(G)** Quantification of telomere length of cells described in (G) [4]. **(H)** Telomere length categories of cells analyzed by telomere DNA FISH in (F, G).; n= number of nuclei analyzed; N, number of telomeres analyzed; average telomere length is indicated by a red bar; numeric average telomere length and standard variation are indicated; a.u.f, arbitrary fluorescence units; an unpaired student's t-test was used to calculate statistical significance.

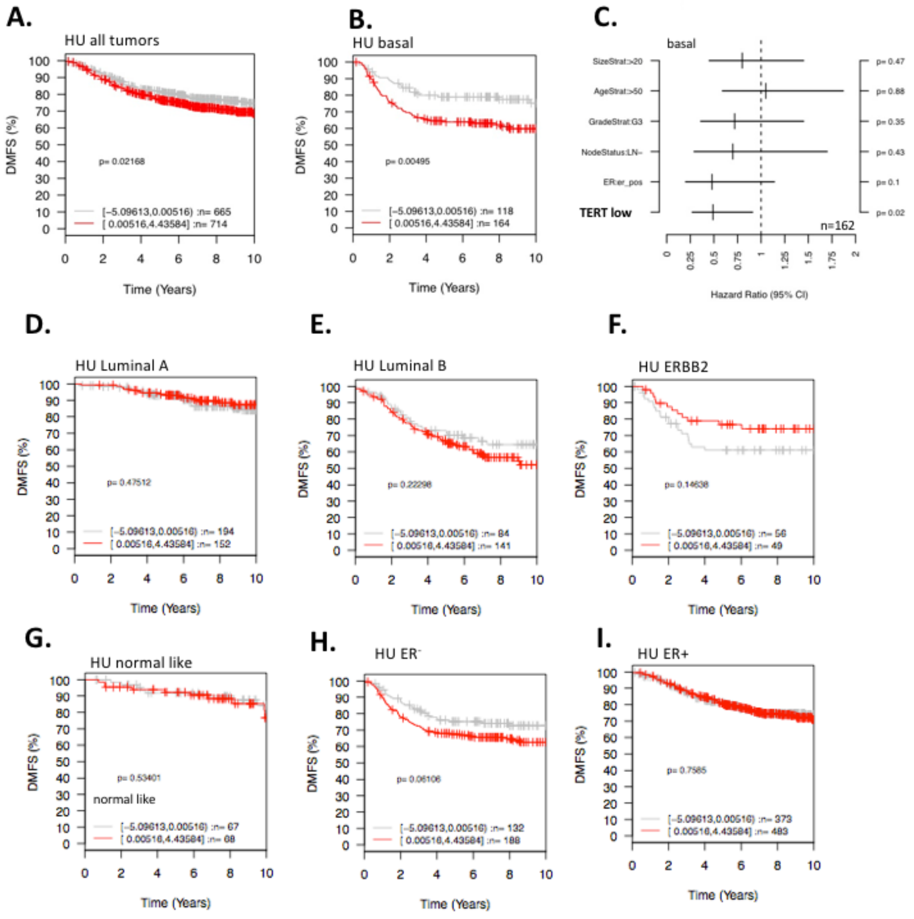

Supplementary Figure 5: **(A-B)** Kaplan–Meier survival curve of time considering both distant metastasis free survival and relapse free survival (DMSF_mixed) of all subtypes of breast cancer (all tumors) and basal type breast cancer (basal type). Patient samples were grouped according to hTERT (Material and methods section). The GOBO tool was used [3]. Red line: high hTERT mRNA expression; grey line: low expression of hTERT. n, number of patients. P-value was calculated by Mantel–Haenszel test. **(C)** Multivariate analysis showing that low hTERT expression behaves as independent predictor of poor clinical outcome in basal type breast cancer in the GOBO dataset [4]. In the panel for each clinical variable and hTERT expression, hazard ratios and corresponding p-value, calculated by cox-regression analysis, are shown. **(D-I)** Kaplan–Meier survival curve of time considering both distant metastasis free survival and relapse free survival (DMSF_mixed) of the indicated breast cancer categories. The GOBO tool was used [3]. Patient samples were grouped according to hTERT (Material and methods section). Red line: high hTERT mRNA expression; grey line: low expression of hTERT. n, number of patients. P-value was calculated by Mantel–Haenszel test. HU is an intrinsic gene expression classifier consisting of a list of genes and a cell proliferation signature that defines breast subtypes [5].

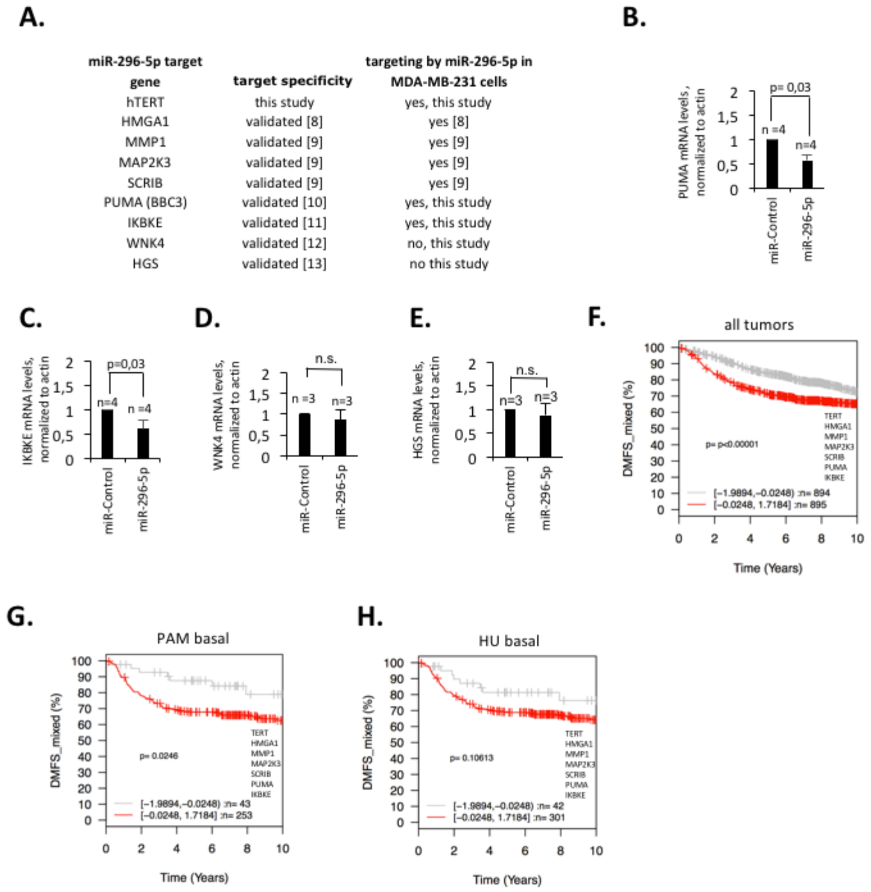

Supplementary Figure 6: (A) List of cancer relevant and experimentally validated miR-296-5p target genes. Candidate target genes that were experimentally confirmed in MDA-MB-231 cells are indicated. **(B-E)** Validation of candidate miR-296-5p target genes in MDA-MB-231 cells. MDA-MB-231 cells were transiently transfected with control or mimic-miR-296-5p; target gene expression was determined by quantitative real-time PCR, 3 days post-transfection. Gene expression was normalized to actin. **(F-H)** Kaplan–Meier survival curve of time considering both distant metastasis free survival and relapse free survival (DMSF_mixed) of the indicated breast cancer categories (GOBO datasets). Patient samples were grouped according to the expression levels of miR-296-5p target genes that were experimentally validated (A, see panels F-H); (Material and methods). Red line: high miR-296-5p target gene expression; grey line: low miR-296-5p target gene expression. HU is a list of genes and a cell proliferation signature that divides breast cancer samples different subtypes [5]; PAM50 is a 50-gene breast cancer subtype predictor [6]. Survival curves: n, number of patients. P-value was calculated using the Mantel–Haenszel test. Quantitative RT-PCR experiments: n, number of independent experiments; error bars show standard deviation, p values in B-E were calculated using a Mann Whitney test; n.s., non significant - p-value >0,05.

A.

miR-512-5p target gene	target specificity	targeting by miR-512-5p MDA-MB-231 cells
hTERT	this study [14]	yes,this study
MCL1	validated [15]	no, this study
CD44	predicted	no, this study
COPZ1	predicted	yes,this study
DBF4	predicted	no, this study
LZTR1	predicted	yes,this study
MAPKAPK3	predicted	no, this study
MN1	predicted	yes,this study

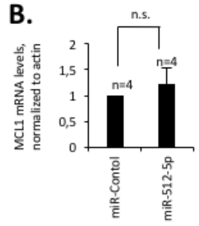

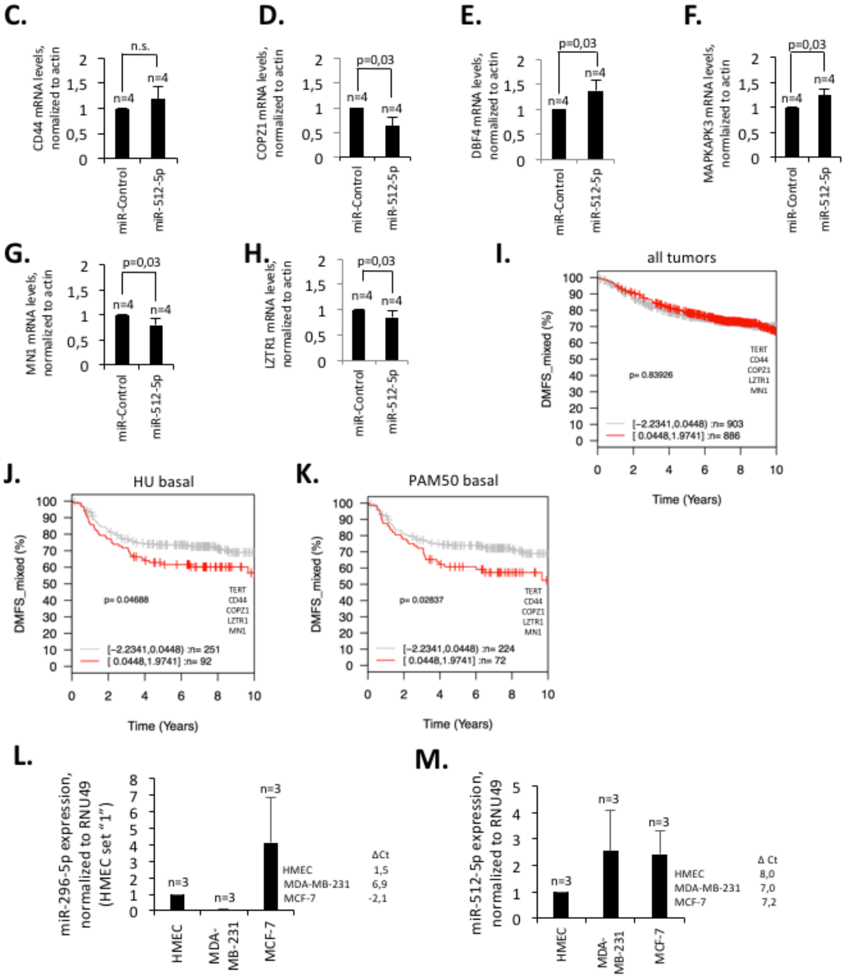

Supplementary Figure 7: (A) List of validated and predicted mir-512-5p target genes that have relevance for human cancer. miRWalk2.0 was used to identify predicted miR-512-5p target gene. Genes were included into the target gene list when 7 out of 9 computational prediction programs suggested miR-512-5p targeting specificity (www.zmf.umm.uni-heidelberg.de/apps/zmf/mirwalk2/index.htmlA). miR-512-5p target genes that were experimentally validated in MDA-MB-231 cells are indicated. **(B-H)** Validation of candidate miR-512-5p target genes in MDA-MB-231 cells. MDA-MB-231 cells were transiently transfected with control or mimic-miR-512-5p ; target gene expression was determined by quantitative real-time PCR, 3 days post-transfection. Gene expression was normalized to actin. **(I-K)** Kaplan–Meier survival curve of time considering both distant metastasis free survival and relapse free survival (DMSF_mixed) of the indicated breast cancer categories (GOBO dataset). Patient samples were grouped according to the expression levels of miR-512-5p target genes validated in MDA-MB-231 cells, listed in (A); (see also Material and methods section). Red line: high miR-512-5p target gene expression; grey line: low miR-512-5p target gene expression; HU is a list of genes and a cell proliferation signature that divides breast cancer samples different subtypes [5]; PAM50 is a 50-gene breast cancer subtype predictor [6]. **(L, M)** miR-296-5p (L) and miR-512-5p (M) expression in human mammary epithelial cells, basal type MDA-MB-231 and luminal type MCF7 cells, as determined by quantitative TaqMan RT-PCR. miRNA levels were quantified against RNU49. ΔCt values (Ct miRNA – Ct RNU49) are indicated. Survival curves: n, number of patients. P-value was calculated using the Mantel–Haenszel test. [5–12]; RT-PCR analysis: n, number of independent experiments; error bars show standard deviation, p values in B-I were calculated using a Mann Whitney test; n.s., non significant - p-value >0,05.

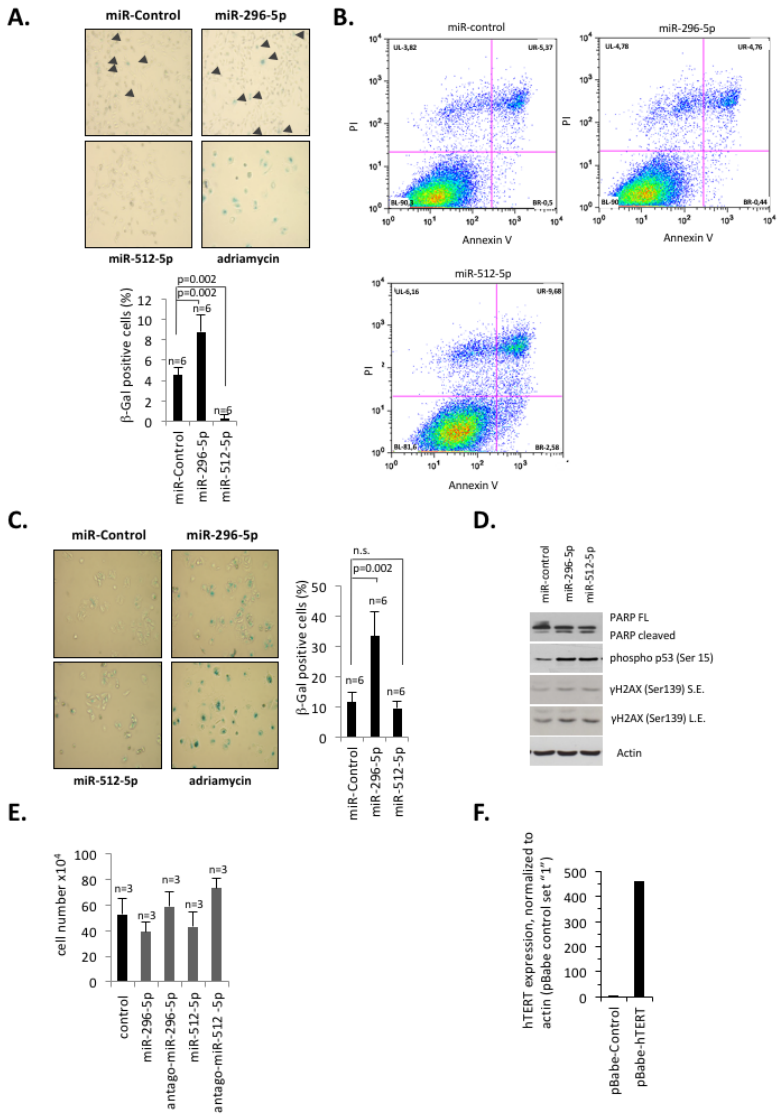

Supplementary Figure 8: (A) β-galactosidase assay of MDA-MB-231 cells transfected with the indicated miRNA mimics. Transfection was carried out twice during the 6 day experimental time window. Treatment of cells with Adriamycin was used as positive control experiment. Bottom panel: quantification of β-galactodisase positive cells. **(B)** FACS analysis of annexin V staining MDA-MB-231 cells transiently transfected with the indicated mimic-miRNA siRNAs. Cells were harvested for detection of Annexin V (apoptotic cells) and PI staining of DNA (necrotic cells). Undamaged live cells resulted as unstained (BL quadrant). Early apoptotic cells, positive to Annexin V only, are shown in the BR quadrant. Late apoptotic cells, positive for both Annexin and PI are shown in the UR quadrant. Necrotic cells, positive to PI only is shown in the UL quadrant. Ectopic miR-296-5p does not show a significant increase in apoptosis rate. **(C)** β-galactosidase assay of MCF-7 cells transfected with the indicated miRNA mimics. Transfection was carried out twice during the 6 day experimental time window. Treatment of cells with Adriamycin was used as positive control experiment. Right panel: quantification of β-galactodisase positive cells. **(D)** Representative images of western blotting experiments using MCF-7 cells transfected with the indicated mimic-miRNAs. Actin was used as a loading control. **(E)** Impact of miR-296-5p and miR-512-5p on MCF-7 breast cancer cell proliferation. Cumulative cell numbers were determined 6 days post-transfection. Cells were transfected with the indicated miRNA mimic or antagomiRs at day 0 and day 3 of the experiment. **(F)** hTERT expression of MDA-MB-231 cells transduced with a retroviral expression vector encoding hTERT, as determined by quantitative RT-PCR. Expression was quantified against actin. hTERT expression of pBabe-control was set 1; n= number of independent experiments; error bars show standard deviation, a Mann Whitney test was used to calculate statistical significance.

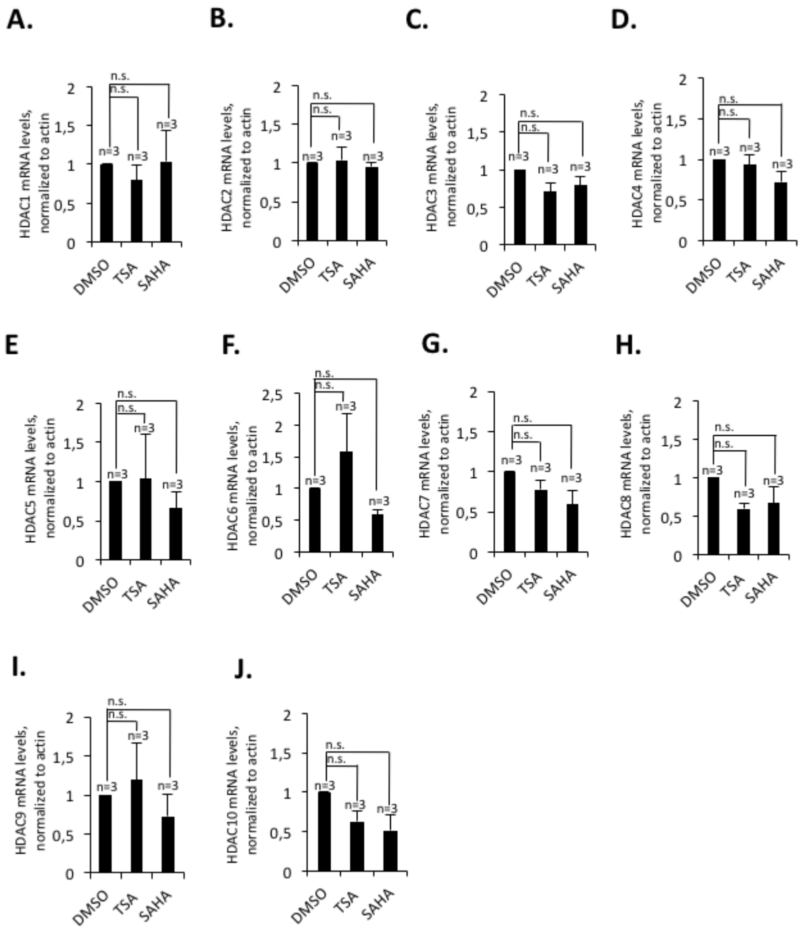

Supplementary Figure 9: (A-J) Gene expression analysis of HDAC1-10. MDA-MB-231 cells were treated with the indicated HDAC inhibitors and expression of HDAC1 (D), HDAC2 (E), HDAC3 (F), HDAC4 (G), HDAC5 (H), HDAC6 (I), HDAC7 (J), HDAC8 (K), HDAC9 (I) or HDAC10 (M) was determined by quantitative real-time PCR. HDAC expression was normalized to actin. RT-PCR analysis: n, number of independent experiments; error bars show standard deviation, p values were calculated using a Mann Whitney test; n.s., non significant - p-value >0,05.

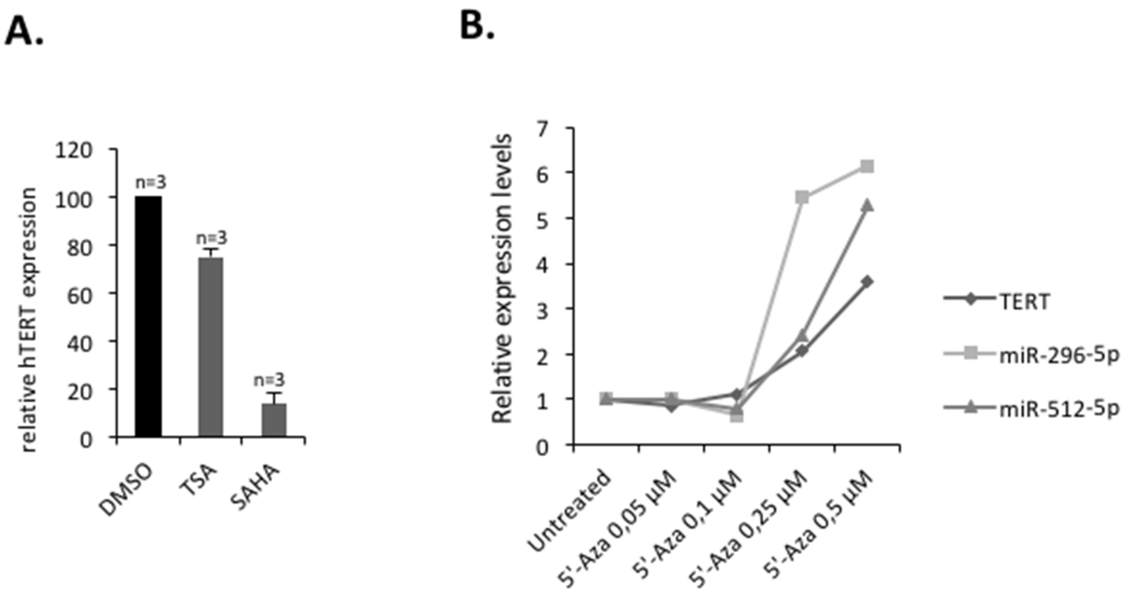

Supplementary Figure 10: (A) hTERT expression of MDA-MB-231 cells with TSA (1 µM) or SAHA (5 µM), as determined by quantitative RT-PCR. hTERT expression was normalized against Actin. **(B)** hTERT and miR-296-5p and miR-512-5p expression after treatment of MDA-MB-231 cells with the indicated concentrations of 5-aza-2'-deoxycytidine (for 4 days), as measured by quantitative RT-PCR. Expression values were quantified against actin (hTERT) or RNU49 (miR-296-5p and miR-512-5p). n= number of independent experiments; error bars show standard deviation; a Mann Whitney test was used to calculate statistical significance.
